# Toward an Integrated Response to Arboviral Threats in the Americas: Perspectives from the Dengue Summit of the Global Health Conference of the Americas 2025

**DOI:** 10.3390/pathogens15101019

**Published:** 2026-09-27

**Authors:** Carlos Espinal, Francisco Becerra-Posada, José Luis Sanmartín, Thais dos Santos, Gamaliel Gutiérrez, Wilfrido Coronell, João Bosco Siqueira, Xavier Sáez-Llorens, Andrea Uboldi, Eder Gatti, Julio Croda, José Alejandro Mojica, Eduardo López-Medina, Zulma Cucunubá, Luis Jorge Hernández, Haroldo Bezerra, Iván Darío Vélez, Mauricio Javier Vera-Soto, Alfonso J. Rodríguez-Morales

**Affiliations:** 1 Global Health Consortium (GHC), Department of Global Health, Robert Stempel College of Public Health & Social Work, Florida International University, Miami, FL 33199, USA; caespina@fiu.edu (C.E.); fbecerra@fiu.edu (F.B.-P.); 2 World Health Organization Regional Office for the Americas/Pan American Health Organization (PAHO), Washington, DC 20037, USA; sanmartj@paho.org (J.L.S.); dosantt@paho.org (T.d.S.); gutierrezg@paho.org (G.G.); bezerrha@paho.org (H.B.); 3 Sociedad Latinoamericana de Infectología Pediátrica (SLIPE), Cartagena 130001, Colombia; wilfridocoronell@gmail.com; 4 Universidade Federal de Goiás, Goiânia 74690-900, Brazil; siqueirajb@gmail.com; 5 Universidad de Panamá, Hospital del Niño Dr. José Renán Esquivel, Senacyt (SNI) y Cevaxin (Vaccine Research Center affiliated to EndPoints Research Sites Network), Panama City 0824, Panama; xsaezll@gmail.com; 6 Secretaria de Salud-Ministerio de Salud, Santa Fe S3000CRJ, Argentina; andreauboldi@gmail.com; 7 Programa Nacional de Inmunizaciones (PNI), Ministry of Health, Brasilia 70058-900, Brazil; eder.gatti@saude.gov.br; 8 Fundação Oswaldo Cruz (Fiocruz), Federal University of Mato Grosso do Sul, Campo Grande 79070-900, Brazil; juliocroda@gmail.com; 9 Programa Ampliado de Inmunizaciones (PAI), Ministry of Health, Bogota 110311, Colombia; jmojica@minsalud.gov.co; 10 Centro de Estudios en Infectología Pediátrica (CEIP), Sociedad Latinoamericana de Infectología Pediátrica (SLIPE), Cali 760043, Colombia; eduardo.lopez@ceiponline.org; 11 Department of Epidemiology and Biostatistics, Pontificia Universidad Javeriana, Bogota 110231, Colombia; zcucunuba@gmail.com; 12 Universidad de los Andes, Bogota 111711, Colombia; luisjorge.hernandezflorez@gmail.com; 13 World Mosquito Program Colombia, Universidad de Antioquia, Medellín 050010, Colombia; idvelez@pecet-colombia.org; 14 Ministerio de Salud y Protección Social, Bogota 110311, Colombia; mvera@minsalud.gov.co; 15 Faculty of Health Sciences, Universidad Científica del Sur, Lima 15074, Peru; 16 Grupo de Investigación Biomedicina, Facultad de Medicina, Fundación Universitaria Autónoma de las Américas-Institución Universitaria Visión de las Américas, Risaralda, Pereira 660003, Colombia

**Keywords:** dengue, arboviruses, Americas, vaccination, *Wolbachia*, surveillance, vector control

## Abstract

Dengue remains the most important arboviral disease in the Americas and a growing global public health threat. The region experienced the largest dengue epidemic in recorded history during 2023–2024, with more than 13 million reported cases in 2024 alone, reflecting the combined effects of rapid urbanization, climate variability, population mobility, persistent social inequities, and weakened vector-control programs. This review summarizes and expands on the principal discussions of the First Dengue Summit of the Dengue Initiative of the Americas, held in Cartagena, Colombia, in September 2025. Major themes included the changing epidemiology of dengue; implementation and development of new-generation vaccines, including TAK-003 and Butantan-DV; advances in digital surveillance, outbreak forecasting, and clinical management; and integrated vector-control strategies. Particular attention is given to *Wolbachia*-mediated population replacement, supported by evidence from a cluster-randomized trial in Indonesia and large-scale quasi-experimental evaluations in Colombia and Brazil, which show substantial and sustained reductions in dengue incidence and hospitalization. In the Aburrá Valley, Colombia, city-wide deployment in Bello, Medellín, and Itagüí was associated with reductions exceeding 90% in notified dengue incidence where *Wolbachia* became established. The review also examines mathematical modeling, artificial intelligence, climate-informed surveillance, and integrated public health approaches. Persistent challenges include financial sustainability, healthcare inequities, climate change, insecticide resistance, and heterogeneous surveillance and health-system capacity. Coordinated, multisectoral, sustainable strategies that integrate vaccination, vector control, surveillance, community participation, and healthcare strengthening are essential to reduce the dengue burden and improve regional resilience.

## 1. Introduction

Dengue is currently the most important arboviral disease affecting the Americas and represents a growing global public health threat. Over the last two decades, the region has experienced an important expansion in dengue transmission driven by rapid urbanization, population growth, climate variability, increased human mobility, weakening vector-control programs, and persistent social inequities [1,2]. The epidemiological landscape has become increasingly complex with the simultaneous circulation of multiple dengue virus serotypes; co-circulation with other arboviruses, including chikungunya virus (CHIKV), Zika virus (ZIKV), Mayaro virus (MAYV), yellow fever virus (YFV), and Oropouche virus (OROV); and recurrent large-scale epidemics affecting both endemic and previously low-transmission areas [3,4,5,6]. In particular, the Americas experienced the largest dengue epidemic in recorded history during 2023–2024, with more than 13 million reported cases in 2024 alone, placing substantial pressure on already fragile healthcare systems [7,8,9].

In response to this evolving scenario, significant advances have emerged in dengue prevention, surveillance, clinical management, mathematical modeling, and vaccine implementation [10,11,12,13,14]. New-generation dengue vaccines, including TAK-003 (now licensed in multiple countries in the Americas and other regions), have demonstrated favorable efficacy and safety profiles and are increasingly being incorporated into national immunization programs [15,16,17,18,19,20,21,22,23,24,25]. Simultaneously, innovative vector control strategies, including *Wolbachia*-based interventions, integrated vector management, and digital surveillance systems, are reshaping public health approaches to arboviral disease prevention [13,26,27,28,29,30,31]. Moreover, advances in clinical management guidelines, critical care protocols, and real-time epidemiological monitoring are contributing to improved preparedness and response capacities across the region [32,33].

Recognizing the urgent need for a coordinated regional response, the first Dengue Summit of the Dengue Initiative of the Americas was convened in Cartagena, Colombia, in September 2025 as a pre-conference event of the Global Health Conference of the Americas, organized by the Global Health Consortium of Florida International University, Miami, FL, USA (https://ghc.fiu.edu/) (accessed on 1 August 2026). The Summit brought together experts from international organizations, academia, health ministries, and research institutions to discuss current challenges and emerging opportunities in dengue prevention and control. This review summarizes and expands on the principal themes discussed at the Summit, focusing on the changing epidemiology of dengue, vaccine implementation, surveillance innovations, vector control strategies, mathematical modeling, and integrated approaches to strengthen regional preparedness and response in the Americas. As a narrative review, we identified relevant literature by searching Web of Science, Scopus, PubMed, SciELO, and ScienceDirect, supplemented by searches of the World Health Organization (WHO) and Pan American Health Organization (PAHO) websites. We also used OpenEvidence and VeraHealth as AI-assisted evidence-discovery tools to identify potentially relevant publications and complement conventional database searching. Queries focused on the review’s principal themes, including dengue epidemiology, vaccination, clinical management, surveillance, mathematical modeling, climate-related risk, and vector-control interventions. We independently checked references identified through these platforms against the original publications before inclusion, and we did not use AI-generated summaries or interpretations in place of assessing the primary sources. Because the objective was to provide a narrative synthesis informed by themes discussed at the Dengue Summit rather than to conduct an exhaustive systematic or scoping review, we did not undertake a PRISMA-based study-selection process or a formal risk-of-bias assessment.

## 2. The Changing Epidemiology of Dengue in the Americas

Dengue epidemiology in the Americas has changed profoundly in recent decades, shifting from sporadic outbreaks to sustained hyperendemic transmission across much of the region. Dengue is now established as the dominant arboviral disease in Latin America and the Caribbean, accounting for the vast majority of reported arboviral infections and imposing a substantial burden on healthcare systems, economies, and vulnerable populations. This transformation reflects the interaction of demographic, environmental, climatic, social, and biological factors that have collectively intensified transmission dynamics throughout the hemisphere [34,35,36,37,38,39,40].

The period between 2023 and 2024 represented a turning point for dengue in the Americas, with the region experiencing the largest epidemic ever recorded, affecting at least 23 countries simultaneously and overwhelming public health systems in multiple settings. Although reported cases declined substantially in 2025 (4.49 million cases) (Table 1), transmission levels remained above historical averages, highlighting the region’s persistent vulnerability to future epidemics (https://www.paho.org/en/arbo-portal/dengue-data-and-analysis) (accessed on 1 August 2026). The widespread geographic distribution of cases demonstrates that dengue is no longer confined to traditionally endemic tropical areas but is increasingly affecting peri-urban, subtropical, and previously low-transmission settings (Table 1) [41,42]. This geographic expansion is being facilitated by rapid urbanization; population mobility; favorable ecological conditions for *Aedes aegypti*, the primary vector of dengue in the Americas; and increasing climate suitability for vector survival and transmission. Areas at the margins of historically endemic zones, including parts of the southern United States, are increasingly experiencing conditions compatible with local transmission and recurrent introduction of dengue viruses. The continental United States provides an illustrative example: in 2024, 105 locally acquired dengue cases were reported, including 85 in Florida, 18 in California, and two in Texas, while travel-associated infections reached record levels. Florida has experienced repeated episodes of autochthonous transmission, including multiple counties in 2024, and locally acquired cases continued to be detected in Miami-Dade County in 2025.

These events remain geographically limited and do not indicate sustained endemic transmission. Still, they demonstrate how repeated viral introductions, widespread competent *Aedes* vectors, high population mobility, and increasingly favorable environmental conditions can create opportunities for local transmission in previously non-endemic settings [43,44,45]. These developments highlight the need to expand preparedness, vector surveillance, diagnostic capacity, and clinician awareness beyond areas traditionally considered endemic [7,46,47].

One of the most concerning epidemiological developments is the simultaneous circulation of all four dengue virus serotypes in several countries in the Americas. Hyperendemic transmission creates favorable conditions for secondary infections, increasing the risk of severe dengue due to immunological mechanisms associated with heterologous infections. Countries such as Brazil, Mexico, Costa Rica, Panama, and Puerto Rico have reported concurrent circulation of multiple serotypes, a phenomenon associated with increased epidemic intensity and more severe clinical presentations. Furthermore, shifts in the age distribution of dengue severity and mortality have been observed across the Americas, although these patterns are heterogeneous between countries and epidemic settings [8]. In some countries, children and adolescents account for an increasing proportion of severe cases and deaths, whereas in others the greatest risk is concentrated among older adults. Brazil provides an important example, where severe dengue and fatal outcomes have been disproportionately observed among older age groups, likely reflecting the combined influence of immunological history, comorbidities, frailty, and reduced physiological reserve [48,49]. These contrasting age-specific patterns underscore the need for national and subnational surveillance systems to routinely stratify dengue burden, hospitalization, severe disease, and mortality by age group and other major vulnerability factors, including pregnancy and relevant comorbidities, rather than assuming a uniform regional risk profile [50,51,52,53,54].

The re-emergence and increasing circulation of DENV-3 in the Americas during 2024–2025 adds another layer of epidemiological complexity. Genomic surveillance has identified much of this resurgence as DENV-3 genotype III, particularly lineage B.3.2 (also referred to within the American II lineage), which has expanded across the Caribbean, Central America, the United States, and South America. Genetically related viruses have been detected in Florida, Puerto Rico, Guatemala, Guyana, Brazil, and, more recently, Paraguay, illustrating extensive regional viral movement and repeated introductions rather than geographically isolated re-emergence. In Paraguay, for example, whole-genome sequencing of DENV-3 detected in 2025 identified genotype III lineage B.3.2 and supported a recent external introduction rather than persistence of previously circulating local strains. This lineage-level resolution is epidemiologically important because large population cohorts in areas with limited recent DENV-3 circulation may have relatively low serotype-specific immunity. Moreover, shifts in serotype and lineage dominance are directly relevant to vaccine-effectiveness assessment and the evaluation of integrated interventions, because observed impact may vary with the circulating viral population. Vaccine and vector-control impact studies should therefore incorporate contemporaneous serotype and genomic surveillance and, where possible, serotype- and lineage-informed outcome analyses [55,56,57,58,59].

Several factors have contributed to the expansion and intensification of dengue transmission in the region. Rapid urbanization, particularly in settings characterized by inadequate housing, deficient water supply systems, and poor waste management, has created ideal ecological conditions for the proliferation of *A. aegypti*. Climate variability and climate change have further influenced vector ecology by expanding mosquito habitats, increasing vector density, and prolonging transmission seasons [60,61,62,63]. Rising temperatures, altered rainfall patterns, and extreme weather events are increasingly recognized as important drivers of arboviral transmission dynamics in the Americas [64,65,66,67]. The COVID-19 pandemic also indirectly worsened the dengue situation [68,69,70,71]. During and after the pandemic, many countries experienced weakened surveillance systems, disrupted vector control activities, reduced access to healthcare, and diverted public health resources toward emergency pandemic response. These disruptions allowed susceptible populations to accumulate and facilitated the resurgence of arboviral transmission once mobility and social interactions normalized [72,73].

Another major challenge involves the co-occurrence of dengue with other arboviral diseases, particularly chikungunya, Zika, Mayaro fever, yellow fever, and Oropouche fever [74]. Pregnancy and the perinatal period deserve particular attention because some of these infections may involve maternal–fetal or perinatal transmission and can result in severe outcomes for fetuses and newborns. The congenital consequences of Zika virus infection are well established [75], while chikungunya virus can be transmitted from mother to newborn when maternal infection occurs close to delivery [76]. More recently, possible vertical transmission of Oropouche virus has emerged as an important concern in the Americas, with reported fetal deaths, pregnancy losses, and congenital abnormalities requiring continued epidemiological and clinical investigation [77,78,79]. These observations reinforce the need to include pregnant women, fetuses, and newborns among priority populations for arboviral surveillance, diagnostic evaluation, and preventive interventions. The overlapping clinical presentations of these infections complicate diagnosis, surveillance, and case management, especially in resource-limited settings [74,77]. Supplementary Table S1 summarizes the principal epidemiological, clinical, laboratory, diagnostic, and preventive characteristics of major arboviral diseases relevant to the Americas. At the same time, increased population mobility, migration and displacement, tourism, and trade continue to facilitate the geographic spread of vectors and viruses across borders, reinforcing the need for coordinated regional surveillance and response mechanisms [80,81].

Beyond the acute phase, dengue may also be followed by persistent symptoms that contribute to a less visible component of disease burden. Post-dengue manifestations can include prolonged fatigue, asthenia, myalgia, arthralgia, headache, sleep disturbances, and impaired physical or cognitive functioning that may persist for weeks or months after the acute infection has resolved [82,83]. Although this post-infectious syndrome remains less well characterized than long COVID [84], the comparison is conceptually useful in highlighting that recovery from dengue is not always complete at the end of the febrile illness. Persistent symptoms may affect return to work or school, quality of life, and healthcare utilization, and their population-level impact may be substantial during large epidemics. Greater attention to standardized definitions, prospective follow-up, and measurement of post-acute outcomes is therefore needed to quantify the full burden of dengue better and inform clinical counseling and health-service planning [82,83].

Given this evolving epidemiological landscape, strengthening preparedness and surveillance systems has become a regional priority. Traditional surveillance approaches that rely solely on passive reporting are increasingly insufficient for anticipating outbreaks in a rapidly changing environment [85]. Integrated surveillance systems that incorporate climate information, genomic monitoring, mathematical modeling, and digital health technologies are becoming essential tools for improving early warning capacity and supporting evidence-based public health decision-making [86,87].

## 3. Advances in Dengue Vaccination and Immunization Strategies

The recent development and implementation of new-generation dengue vaccines represent one of the most important advances in arboviral disease prevention in the Americas. After decades of limited preventive options focused primarily on vector control and clinical management, vaccination has emerged as a promising complementary strategy to reduce dengue morbidity, hospitalizations, and mortality in endemic regions (Table 2) [11,16,21,23].

The increasing frequency and magnitude of epidemics in Latin America and the Caribbean have accelerated interest in vaccine implementation, particularly following the unprecedented regional epidemic observed during 2023–2024 [18,21,22,23,41,88]. Table 2 summarizes the principal complementary strategies currently available for integrated dengue prevention and control in the Americas.

The dengue vaccine landscape has evolved substantially, with available and recently licensed vaccines differing in platform, dosing schedule, dependence on previous dengue exposure, serotype-specific efficacy, and programmatic considerations (Table 3). Among currently available vaccines, TAK-003 has attracted substantial attention due to its favorable efficacy and safety profile demonstrated in clinical trials and early real-world implementation studies [15,16,19,20,21,24,89].

The vaccine has shown sustained protection against symptomatic dengue and hospitalization over several years of follow-up, with particularly strong protection against severe disease. Real-world effectiveness data from the Americas increasingly complement clinical-trial findings. During the 2024 dengue outbreak in São Paulo State, Brazil, a test-negative study among adolescents aged 10–14 years estimated TAK-003 effectiveness against symptomatic, virologically confirmed dengue at 50.2% (95%CI 45.0–54.9) after the first dose and 61.7% (95%CI 39.9–75.6) after the second dose, while first-dose effectiveness against dengue hospitalization was 67.5% (95%CI 43.4–81.3). Notably, effectiveness against symptomatic disease was 67.4% (95%CI, 57.2–75.1) at 14–27 days after the first dose but declined to 49.7% (95%CI, 30.4–63.6) beyond 90 days.

This early decline in protection after a single dose has important programmatic implications, particularly for completing the two-dose schedule and for strategies that consider vaccination in epidemic or emergency-response settings [22,90,91]. Importantly, the safety experience with TAK-003 differs from that of CYD-TDV, for which an increased risk of hospitalized and severe dengue among previously seronegative recipients became a major safety and policy concern. To date, no comparable safety signal has been demonstrated for TAK-003; however, the available evidence does not exclude differential risk across serotypes or baseline serostatus, particularly for DENV-3 and DENV-4 in seronegative individuals, for whom case numbers remain limited, and confidence intervals are wide. Continued long-term, serotype-specific safety surveillance is therefore warranted [92]. These findings have contributed to growing confidence among public health authorities and technical advisory groups considering vaccine introduction in endemic settings [15,16,19,20,21,24,93,94].

Another important development in the regional vaccine landscape is Butantan-DV, a live-attenuated tetravalent vaccine derived from the TV003 platform and developed through collaboration between Instituto Butantan and the U.S. National Institutes of Health. Unlike the two-dose TAK-003 schedule, Butantan-DV is administered as a single dose and has demonstrated sustained efficacy through five years of follow-up in its phase 3 trial among participants aged 2–59 years. Overall vaccine efficacy against symptomatic, virologically confirmed dengue was 65.0%, including 58.9% among participants without evidence of previous dengue exposure, while efficacy against severe dengue or dengue with warning signs was 80.5%. These findings are particularly relevant because they demonstrate protection in both dengue-experienced and dengue-naive populations while offering the programmatic advantage of a single-dose schedule. Butantan-DV is also strategically important for the Americas because it represents a vaccine developed and manufactured within the region, strengthening regional production capacity and potentially contributing to greater autonomy and resilience in vaccine supply. Brazil granted regulatory approval in 2025, although large-scale implementation, effectiveness, and post-marketing safety evaluation remain evolving (Table 3 and Table 4) [22,90,91].

International recommendations now support the use of TAK-003 in countries with high dengue transmission intensity, particularly among children and adolescents living in endemic areas (https://iris.who.int/server/api/core/bitstreams/451d6f6a-c534-4969-b67e-b8d92b8d03c4/content) (accessed on 1 August 2026). One major operational advantage of this vaccine is that it can be administered without pre-vaccination serological screening, simplifying implementation and reducing logistical barriers. This feature is especially important in low- and middle-income countries where laboratory infrastructure and financial resources may limit large-scale screening strategies [11,92,95,96].

Several countries in the Americas have already initiated dengue vaccine implementation efforts or vaccination programs, although regulatory indications and programmatic strategies vary substantially across countries (Table 4) [97,98]. Brazil offers one of the most informative examples of the opportunities and constraints of large-scale dengue vaccination. In February 2024, Dourados, Mato Grosso do Sul, launched the world’s first mass dengue vaccination campaign, targeting approximately 120,000 individuals aged 4–60 years with TAK-003. This broad age-based municipal strategy contrasted with the contemporaneous national rollout through the *Programa Nacional de Imunizações* (National Immunization Program), which initially prioritized individuals aged 10–14 years in selected high-transmission municipalities because vaccine supply was insufficient to support broader population coverage. The Brazilian experience therefore illustrates a central operational tension for dengue vaccination programs: regulatory indications and epidemiological evidence may support vaccination across wider age groups, while real-world procurement and supply constraints can necessitate substantially narrower prioritization. These constraints affect equity, population-level impact, schedule completion, and how vaccine-effectiveness studies are interpreted. Baseline epidemiological characterization is also essential for evaluating impact; an 11-year analysis of Dourados and Ponta Porã documented marked temporal and age-specific variation in dengue incidence before vaccine introduction and provides an important reference for subsequent effectiveness assessments [99].

Post-marketing surveillance in Dourados also provides important operational safety data for implementing TAK-003. Among 124,483 administered doses, 88 adverse events following immunization (AEFI) were reported, corresponding to 70.69 events per 100,000 doses, while serious AEFI were uncommon at 6.42 per 100,000 doses. Importantly, 27 reported events, representing 30.68% of all AEFI, were classified as immunization errors, frequently related to gaps in training. These findings reinforce that vaccine safety during large-scale introduction depends not only on pharmacovigilance for biological adverse events but also on adequate workforce preparation, standardized administration procedures, and rapid identification of programmatic errors. Countries introducing dengue vaccination should therefore integrate active safety surveillance with structured training and quality-assurance mechanisms from the outset [100,101,102].

Argentina has adopted a phased, adaptive vaccination strategy that prioritizes areas with the highest dengue burden based on epidemiological, geographic, socioeconomic, and healthcare-access criteria, with progressive implementation guided by local epidemiology and operational capacity [98,103,104]. Initial phases prioritized controlled vaccination activities within healthcare facilities to improve staff training, monitor adverse events, and address vaccine-related anxiety before broader community deployment. Subsequent outreach strategies included mobile vaccination units and community-based interventions designed to increase access in vulnerable populations. This gradual approach highlighted the importance of flexibility, operational learning, and local adaptation during the early stages of vaccine implementation [105].

At the time of writing, several countries in the region were at different stages of vaccine introduction. Different countries in the region have advanced discussions regarding vaccine introduction and implementation, particularly in municipalities with high seroprevalence and recurrent epidemic transmission. Modeling studies suggest that targeted vaccination strategies in high-transmission settings may provide the greatest epidemiological impact and cost-effectiveness [106]. However, these analyses also underscore important uncertainties, including variability in serotype-specific effectiveness, long-term protection, and local epidemiological heterogeneity. Consequently, vaccine introduction decisions require careful evaluation of local transmission dynamics, age-specific burden, healthcare capacity, and economic considerations [107].

Large-scale dengue vaccination in the Americas still faces important programmatic and financial constraints. Financial sustainability remains a principal barrier to large-scale dengue vaccination in the Americas. Vaccine introduction requires substantial upfront and recurrent investment for procurement, delivery, cold-chain infrastructure, surveillance, pharmacovigilance, communication, and program evaluation. At the same time, many endemic countries must allocate limited public budgets across multiple competing health priorities. Consequently, decisions regarding vaccine introduction and expansion should incorporate not only epidemiological impact and cost-effectiveness but also affordability, budget impact, financing mechanisms, and long-term program sustainability [108,109]. In addition, implementation strategies must account for complex epidemiological patterns that vary significantly across countries and even between neighboring regions within the same country. Local seroprevalence, transmission intensity, population age structure, and healthcare access all influence the potential impact of vaccination programs [110,111].

Communication and community engagement, informed by population-level assessments of knowledge, attitudes, practices, and vaccine acceptance, have emerged as critical components of successful implementation [112]. The experience with CYD-TDV (Dengvaxia^®^), particularly the controversy that followed its large-scale use in the Philippines, remains a defining episode in dengue vaccination policy and risk communication. Subsequent evidence that seronegative vaccine recipients could face an increased risk of severe dengue and hospitalization led to major changes in recommendations, restrictions on use, and prolonged effects on public confidence in vaccination programs (https://www.who.int/groups/global-advisory-committee-on-vaccine-safety/topics/dengue-vaccines/safety-in-philippines) (accessed on 1 August 2026) [113]. This experience showed how quickly safety concerns can shape regulatory policy, political debate, media narratives, and community trust, and it continues to influence the cautious approach to newer dengue vaccines [113]. Effective vaccination programs therefore require transparent communication strategies, continuous community engagement, and strong pharmacovigilance systems that can rapidly identify and respond to safety concerns. Communication strategies should also explicitly address misinformation and disinformation through proactive risk communication, social listening to identify emerging concerns, rapid correction of false or misleading claims, and dissemination of clear, consistent, and locally adapted messages through trusted healthcare professionals, community leaders, schools, and other credible local actors [114,115]. Communication should begin before implementation, rather than only after controversy emerges, and clearly explain expected benefits, uncertainties, adverse-event monitoring, and the rationale for public health recommendations. Building confidence among healthcare workers, policymakers, and communities remains essential for achieving high vaccination coverage and long-term program sustainability [116,117].

Another important consideration is integrating vaccination into broader dengue prevention frameworks. Vaccines alone are unlikely to eliminate dengue transmission in highly endemic settings characterized by intense vector circulation and complex ecological drivers. Instead, vaccination should be viewed as one component of a comprehensive strategy that also includes vector control, strengthened surveillance systems, early outbreak detection, improved clinical management, and community participation. Integrated approaches are particularly important in the context of climate change and expanding arboviral threats, where adaptive and multisectoral public health responses are increasingly necessary [118,119].

The growing experience with dengue vaccine implementation in the Americas provides valuable lessons for the future. Operational experiences from Brazil and Argentina, together with targeted implementation and pilot vaccination programs in countries such as Peru, demonstrate the feasibility of introducing dengue vaccination through different programmatic models when supported by political commitment, technical expertise, surveillance infrastructure, and community engagement. At the same time, continued evaluation is needed to characterize short- and long-term effectiveness, post-dose protection, completion of multidose schedules, duration of protection, potential booster requirements, and effectiveness under changing serotype distributions. As dengue continues to expand throughout the region, vaccination is likely to become an increasingly important component of integrated public health strategies to reduce the burden of arboviral diseases in the Americas [120,121].

## 4. Mathematical Modeling, Forecasting, and Decision-Making Tools

The increasing complexity of dengue transmission dynamics in the Americas has highlighted the importance of mathematical modeling and predictive tools for improving outbreak preparedness, resource allocation, and public health decision-making [50,122,123]. Traditional surveillance systems that rely exclusively on passive case reporting are often insufficient to anticipate epidemic waves in rapidly changing epidemiological scenarios shaped by climate variability, urbanization, population mobility, and the simultaneous circulation of multiple dengue serotypes. Consequently, the incorporation of advanced modeling approaches has become increasingly relevant for guiding prevention and control strategies [41,52,124].

Mathematical models have played a central role in evaluating the potential impact of dengue vaccination programs. However, dengue presents unique modeling challenges because its multifactorial transmission system involves interactions among humans, mosquito vectors, environmental conditions, immunity, and four antigenically distinct viral serotypes [125,126,127]. Additional complexities include heterogeneous transmission intensities across regions, imperfect surveillance systems, age-dependent immunity patterns, and uncertainties about vaccine-induced protection. These factors make long-term predictions difficult and require models that integrate epidemiological, demographic, immunological, and ecological variables [128,129,130].

Recent modeling studies in the Americas have focused on identifying geographic areas with the highest potential benefit from vaccination. Seroprevalence-based analyses and transmission models have helped prioritize municipalities and population groups in which vaccine implementation may yield the greatest epidemiological impact and cost-effectiveness [131,132]. These approaches are particularly important in resource-limited settings where phased or targeted vaccination strategies may be necessary. Modeling can also help ministries of health evaluate different implementation scenarios, estimate reductions in hospitalizations, and project future disease burden under varying intervention strategies [133,134].

In parallel, advances in artificial intelligence and machine learning have introduced new opportunities for epidemic forecasting [135,136,137]. Long Short-Term Memory neural networks and other predictive algorithms are increasingly being applied to dengue surveillance data to identify epidemiological trends and provide early outbreak warnings [138,139,140]. These systems analyze large historical datasets to detect temporal patterns and generate short-term predictions regarding transmission dynamics. Although these models are generally more effective at identifying trends than predicting exact case numbers, they offer valuable support for public health preparedness and operational planning [124].

Integrating climate and environmental information into predictive systems represents another important advance. Temperature, rainfall, humidity, and extreme weather events strongly influence mosquito ecology and viral transmission, making climate-sensitive forecasting models increasingly useful in arboviral surveillance. Combined with genomic surveillance, mobility data, and real-time epidemiological reporting, these tools may improve early warning capacities and strengthen regional preparedness for future outbreaks [141,142,143].

Modeling and forecasting systems remain constrained by incomplete or delayed reporting, heterogeneous surveillance quality, limited availability of high-resolution data, and uncertainties surrounding serotype-specific vaccine effectiveness and long-term immunity. Their greatest value, therefore, lies not in producing deterministic predictions but in supporting scenario analysis, early warning, resource allocation, and adaptive decision-making within continuously updated surveillance systems. Many countries still experience incomplete or delayed reporting, variable surveillance quality, and limited access to high-resolution epidemiological data. In addition, uncertainty regarding vaccine effectiveness against specific serotypes and long-term immunity complicates predictive analyses. Nevertheless, mathematical modeling and digital forecasting tools are becoming essential components of modern dengue prevention strategies, supporting evidence-based decision-making and enabling more adaptive, proactive public health responses across the Americas [125,144].

## 5. Innovations in Surveillance and Clinical Management

The growing burden of dengue and other arboviral diseases in the Americas has accelerated the development of innovative surveillance systems and strengthened clinical management strategies across the region. Recent epidemics have shown the limits of traditional public health approaches, especially in settings with fragmented reporting, delayed outbreak detection, and insufficient healthcare preparedness. As a result, regional and international organizations have promoted integrating digital surveillance platforms, real-time data analysis, standardized clinical guidelines, and capacity-building initiatives to improve epidemic response and patient outcomes [39,145,146].

One of the most important advances has been the expansion of integrated arboviral surveillance platforms that can consolidate epidemiological information from multiple countries in near real time. These systems incorporate interactive dashboards, automated reporting tools, geographic information systems, and data visualization technologies that facilitate rapid interpretation of epidemiological trends. Modern surveillance infrastructure increasingly combines laboratory and point-of-care diagnostic data, clinical case notifications, vector surveillance, climate information, and outbreak monitoring into unified digital platforms that support evidence-based public health decision-making [147,148].

Diagnostic capacity is a critical component of integrated arboviral surveillance, particularly in settings where dengue and other arboviral diseases, including chikungunya, Zika, and Oropouche fever, co-occur and have overlapping clinical presentations. Rapid NS1 antigen assays can support early dengue diagnosis in the first days of illness, especially where molecular testing is not immediately available. However, sensitivity varies with timing, serotype, immune status, and assay characteristics. RT-PCR remains the preferred method for etiologic confirmation during the viremic phase and, where available, multiplex molecular platforms can simultaneously test for several arboviruses, improving differential diagnosis and reducing delays in outbreak recognition. Point-of-care molecular and antigen-based technologies may further expand diagnostic access in emergency departments, primary care facilities, and remote settings. However, implementation requires attention to assay validation, quality assurance, cost, specimen timing, and integration of results into real-time surveillance systems. Strengthening decentralized diagnostic networks and referral pathways to reference laboratories should therefore be considered a core element of regional arboviral preparedness [149,150].

Regional initiatives have also strengthened collaboration and information sharing among countries through virtual cooperation networks and digital communication platforms. These mechanisms facilitate real-time exchange of technical expertise, outbreak alerts, surveillance methodologies, and clinical experiences between ministries of health, research institutions, and public health professionals. Virtual cooperation spaces have become particularly valuable for countries with limited specialized expertise, enabling rapid dissemination of best practices and promoting more standardized regional responses to arboviral threats [151,152].

Another important innovation involves incorporating predictive analytics and automated reporting systems into routine surveillance activities. Several platforms now generate daily epidemiological updates and interactive visualizations using automated programming systems connected directly to national databases. These tools improve the timeliness of outbreak detection and provide health authorities with updated information necessary for operational planning and emergency response. In addition, the integration of climate and environmental data into surveillance systems is enhancing the ability to identify conditions favorable for vector proliferation and increased transmission risk [86,146,153].

At the clinical level, significant progress has been achieved through the development and implementation of evidence-based guidelines for dengue management (https://iris.paho.org/handle/10665.2/55867) (accessed on 1 August 2026). Standardized clinical protocols have become increasingly important given the large number of severe cases observed during recent epidemics and the persistent variability in clinical practices across healthcare settings. Updated guidelines emphasize systematic patient assessment, early recognition of warning signs, timely fluid management, and risk stratification to reduce morbidity and mortality associated with severe dengue (https://www.who.int/publications/i/item/9789240111110) (accessed on 1 August 2026). Risk stratification should also explicitly account for populations at greater risk of severe disease or adverse outcomes. Neonates and young infants may present atypically and have limited physiological reserve; pregnant women require careful assessment because dengue may be associated with maternal complications, bleeding, preterm delivery, fetal compromise, and perinatal transmission when infection occurs close to delivery. Older adults are at increased risk of hospitalization, severe dengue, organ dysfunction, and death, particularly in the presence of frailty and chronic disease. Comorbidities such as diabetes mellitus, cardiovascular disease, chronic kidney disease, obesity, and other conditions that impair physiological reserve may further increase the risk of clinical deterioration and complicate fluid management. These groups therefore require lower thresholds for close monitoring, timely referral, and escalation of care when warning signs or organ dysfunction emerge [154,155,156].

Although antiviral therapies were not a focus of the Dengue Summit, developing dengue-specific therapeutics is an important emerging area. At present, no antiviral or host-directed pharmacological treatment has been approved for routine dengue management, which remains primarily supportive. Nevertheless, several therapeutic strategies are under investigation, including direct-acting antivirals targeting viral proteins such as NS4B, NS5 polymerase, and NS3 protease, as well as host-directed agents and monoclonal antibodies. Early clinical studies have provided proof of concept for antiviral activity in humans, but evidence remains insufficient to support routine use. Continued clinical development will require well-designed trials evaluating both virological and clinically meaningful outcomes [157,158].

Particularly noteworthy has been the development of specialized guidelines for managing severe dengue in intensive care units. These recommendations were developed using rigorous, evidence-based methodologies and multidisciplinary collaboration among experts in infectious diseases, pediatrics, intensive care, emergency medicine, and public health. The guidelines provide structured algorithms for patient classification, hemodynamic monitoring, shock management, and organ support, helping standardize care for critically ill patients across the region [159,160].

Educational and capacity-building initiatives have complemented these clinical advances. Large-scale online training programs, regional workshops, technical missions, and hybrid educational models have trained hundreds of thousands of healthcare professionals in dengue diagnosis and management [161,162]. These efforts are particularly important in endemic countries where recurrent epidemics place substantial pressure on frontline healthcare workers and hospital systems, especially in settings with limited specialist availability and constrained referral and critical care capacity [163,164].

Important gaps in access to timely and appropriate dengue care remain across the region. Many healthcare systems in the Americas continue to face shortages of adequately trained personnel, limited availability of critical care beds, and unequal access to diagnostic and monitoring technologies, particularly in rural, remote, and underserved areas. These limitations can delay recognition of warning signs, timely referral, hemodynamic monitoring, and escalation of care in patients with severe dengue, thereby increasing the risk of preventable complications and death. Strengthening referral networks, emergency transport systems, frontline training, and surge capacity for critical care should therefore be considered essential components of dengue preparedness. In addition, surveillance quality remains heterogeneous between countries, limiting the comparability of epidemiological data and complicating regional analyses. Closing these gaps will require sustained investment in workforce development, referral networks, critical-care capacity, diagnostic access, and regional coordination, particularly in rural and underserved settings [165,166,167].

## 6. Vector Control and Integrated Prevention Strategies

The sustained expansion of dengue transmission in the Americas has reinforced the critical importance of vector control as a central component of arboviral disease prevention. Despite advances in vaccination and surveillance, reducing human exposure to *Aedes* mosquitoes remains essential for limiting transmission, particularly in highly endemic urban environments. However, recent epidemics have demonstrated that conventional vector control strategies alone are insufficient to contain large-scale outbreaks, especially in settings characterized by rapid urbanization, climate variability, and persistent socioeconomic inequalities. Consequently, integrated and innovative prevention approaches are increasingly being prioritized across the region [168,169,170].

*A. aegypti* remains highly adapted to urban and peri-urban environments throughout Latin America and the Caribbean. Inadequate water storage systems, poor waste management, overcrowded housing, and irregular access to public services create ideal breeding conditions for mosquito proliferation. In many cities, persistent informal settlements and poor urban infrastructure significantly complicate vector control activities. Importantly, dengue transmission should not be conceptualized as occurring predominantly within households. People also come into contact with vectors in places where they spend long periods or congregate, including schools, workplaces, markets, transportation hubs, and healthcare facilities. Consequently, vector surveillance and control strategies should extend beyond residential premises to incorporate these socially important transmission environments, particularly during periods of intense community transmission [171,172]. Furthermore, asymptomatic and paucisymptomatic dengue infections contribute substantially to silent viral circulation, allowing for transmission to persist even during periods when clinically apparent disease and routine case notifications appear low. This hidden component of transmission can delay recognition of increasing community-level viral activity and reinforces the need to complement passive clinical surveillance with laboratory, entomological, and other early-warning approaches [173,174,175].

Traditional vector control programs have faced multiple operational challenges in recent years. Several countries saw mosquito control activities weaken during and after the COVID-19 pandemic because of resource diversion, workforce shortages, and disruptions to routine public health operations. Insecticide resistance has emerged as an increasing concern, progressively reducing the effectiveness of conventional chemical control in some endemic settings and reinforcing the need for routine resistance monitoring, rotation of insecticides with different modes of action, and evaluation of alternative formulations and complementary control tools. At the same time, repeated reliance on community mobilization to eliminate breeding sites has created an important operational challenge. Community participation is often strongest during periods of intense transmission but declines once perceived risk decreases, producing cycles of engagement and disengagement that undermine the continuity of source-reduction activities. Periodic appeals by health authorities to eliminate containers and other breeding sites may therefore have limited impact when they are not accompanied by sustained education, environmental management, municipal services, monitoring, and appropriate regulatory or enforcement mechanisms [176,177,178,179].

These limitations reinforce the importance of the Integrated Vector Management (IVM) approach promoted by WHO and PAHO, which emphasizes coordinated, evidence-based, context-specific interventions rather than reliance on a single control measure. IVM combines entomological surveillance and insecticide-resistance monitoring, environmental management, chemical and biological control, community participation, regulatory measures where appropriate, intersectoral collaboration, and continuous evaluation of intervention effectiveness. Within this framework, decisions regarding vector control technologies should be guided by local transmission patterns, insecticide susceptibility, operational feasibility, cost, sustainability, and equity considerations [180,181].

Among innovative vector-control approaches, *Wolbachia*-mediated population replacement has accumulated particularly strong epidemiological evidence for sustained reduction in dengue transmission at community scale (https://www.who.int/news/item/11-06-2026-who-update-on-the-assessment-of-wolbachia-population-replacement-for-the-control-of-aedes-borne-diseases) (accessed on 1 August 2026). *Wolbachia* is a naturally occurring intracellular bacterium that, when stably introduced into *A. aegypti* populations, substantially reduces mosquito competence for dengue and several other arboviruses.

*Wolbachia* is a naturally occurring intracellular bacterium that does not naturally infect *A. aegypti*. The *w*Mel strain used for population replacement was artificially introduced into *A. aegypti* through embryonic microinjection and is subsequently maternally transmitted. Its public health utility derives from two complementary biological properties: *w*Mel substantially reduces mosquito vector competence for DENV and several other arboviruses and induces cytoplasmic incompatibility. Matings between *w*Mel-infected males and uninfected females generally fail to produce viable offspring, whereas *w*Mel-infected females can reproduce successfully with both infected and uninfected males. This reproductive advantage, together with maternal transmission, facilitates the spread and stable establishment of *w*Mel within *A. aegypti* populations, progressively replacing the wild-type population with mosquitoes having reduced competence for arbovirus transmission [182].

In a cluster-randomized controlled trial in Yogyakarta, Indonesia, deploying *w*Mel-infected *A. aegypti* reduced virologically confirmed symptomatic dengue by 77.1% and dengue-related hospitalizations by 86.2%, with protection observed across all four dengue virus serotypes. These findings are supported by large-scale observational and quasi-experimental evaluations in Colombia and Brazil showing substantial, sustained reductions in dengue incidence following successful *Wolbachia* establishment. Beyond dengue, experimental vector-competence studies indicate that *w*Mel can also inhibit transmission of Zika virus, chikungunya virus, Mayaro virus, and yellow fever virus, suggesting a potentially important multipathogen benefit in areas where these arboviruses co-circulate. However, this broader protective effect is supported predominantly by laboratory evidence and should not yet be considered equivalent to the population-level epidemiological evidence established for dengue [26,27,28,29,30,31,183,184].

Evidence from Brazil further supports the effectiveness and scalability of *Wolbachia*-mediated population replacement. In Niterói, Rio de Janeiro State, phased deployment during 2017–2019 was initially associated with a 69.4% reduction in notified dengue incidence compared with untreated areas; subsequent city-wide follow-up demonstrated durable *Wolbachia* establishment and an 88.8% reduction in notified dengue incidence relative to the pre-intervention period, including sustained protection during the exceptionally intense 2024 epidemic. In Campo Grande, Mato Grosso do Sul, researchers released more than 100 million *Wolbachia*-infected mosquitoes between 2020 and 2023 as part of a large-scale, government-supported deployment. Stable establishment of *w*Mel at ≥60% prevalence was associated with a 63.2% reduction in dengue incidence. Together with randomized evidence from Yogyakarta and the large-scale Colombian experience, these findings demonstrate reproducible epidemiological effectiveness across distinct urban settings and support the transition of *Wolbachia* from research-led deployment to routine public health implementation [183,185,186].

This potential multipathogen effect is particularly relevant to integrated arboviral control in the Americas. Laboratory studies have shown strong *w*Mel-mediated inhibition of Zika virus in *A. aegypti*, including reduced transmission potential, and experimental work also supports inhibition of Mayaro virus. Because no licensed vaccines are currently available against ZIKV or MAYV, reduction in vector competence could provide an additional public health benefit where these viruses overlap geographically with DENV. Experimental evidence also supports inhibition of yellow fever virus, raising the possibility that established *Wolbachia*-carrying *A. aegypti* populations could provide an additional barrier to urban yellow fever transmission. Such an effect, however, should be regarded strictly as complementary to, and never a replacement for, yellow fever vaccination [187,188].

Beyond evidence of epidemiological effectiveness, *Wolbachia*-mediated population replacement has also reached a substantial degree of operational maturity. Its biological mechanisms, community engagement approaches, mass-rearing systems, egg- and adult-release procedures, entomological monitoring, establishment criteria, and epidemiological evaluation methods have now been implemented and standardized across multiple settings. This accumulated operational experience supports the view that *Wolbachia* should be considered not merely as an experimental technology but as a scalable public health intervention that can be incorporated into routine dengue-control programs where epidemiological suitability, financing, implementation capacity, and community acceptance are adequate [27,189,190]. The Aburrá Valley experience in Colombia provides an important example of implementation at metropolitan scale. Deployment across Bello, Medellín, and Itagüí achieved stable *Wolbachia* establishment across a population of more than 3 million people. It was associated with reductions exceeding 90% in notified dengue incidence in areas where *Wolbachia* became established. Beyond the epidemiological effect, the Colombian program demonstrated the feasibility of large-scale local egg production, quality-assurance systems, staged community engagement, geographically intensive release operations, and sustained post-release entomological and epidemiological surveillance. These operational components now constitute a transferable implementation framework that can be adapted to national and subnational dengue-control programs rather than being regarded as unresolved technical barriers [26,27,185].

Global implementation has also progressed substantially. By 2026, *Wolbachia* population-replacement programs had been implemented in 15 countries, protecting more than 16 million people. Brazil has moved from demonstration projects to programmatic scale-up under the Ministry of Health’s leadership, supported by expanded national production capacity and implementation across multiple municipalities. WHO’s Vector Control Advisory Group concluded in 2020 that *Wolbachia* population replacement had demonstrated epidemiological impact and advised initiating the guideline development process (https://www.who.int/publications/b/54695) (accessed on 1 August 2026). WHO is currently updating the evidence base, developing an operational manual, and preparing formal guideline consideration for population replacement against *Aedes*-borne diseases (https://www.who.int/news/item/11-06-2026-who-update-on-the-assessment-of-wolbachia-population-replacement-for-the-control-of-aedes-borne-diseases) (accessed on 1 August 2026). Together, these developments support a transition from repeated pilot projects toward planned, adequately financed, large-scale implementation within integrated national dengue-control strategies (https://www.worldmosquitoprogram.org/stories/annual-review-2025) (accessed on 1 August 2026) [191].

One of the principal advantages of *Wolbachia*-based population replacement is its potential for long-term persistence after successful introgression, reducing the need for continuous releases. However, establishment and persistence vary across settings. In Niterói, early post-release monitoring showed heterogeneous *w*Mel prevalence, with some release zones maintaining median frequencies of about 40–70%, although subsequent city-wide implementation achieved high, durable prevalence. Similarly, in Campo Grande, 89% of intervention areas achieved stable *w*Mel prevalence ≥ 60%, indicating that a minority of areas required longer introgression periods or did not reach the predefined threshold for stable establishment. Environmental conditions may also affect persistence, as high-temperature exposure and heatwaves can temporarily reduce *w*Mel density and infection frequency. Long-term entomological monitoring and, where necessary, supplementary releases should therefore remain integral components of implementation programs [185]. Economic evaluations have suggested favorable cost-effectiveness for *Wolbachia* deployment in high-density urban settings, with modeling analyses estimating gross cost-effectiveness below approximately US$1500 per disability-adjusted life year (DALY) averted and the potential for cost savings over longer time horizons when healthcare and societal costs averted are considered [192]. Nevertheless, the substantial upfront investment required for large-scale *Wolbachia* deployment, together with the need for sustained financial, operational, and political support during implementation and monitoring, may limit its accessibility in resource-constrained settings and potentially widen inequities in access to innovative vector-control technologies. Therefore, *Wolbachia* should not be regarded as a stand-alone intervention but rather as one component of an IVM strategy adapted to local epidemiological, entomological, financial, and operational conditions. Several implementation settings have documented high community acceptance when deployment was preceded by structured engagement and transparent communication; for example, pre-release acceptance in the Colombian cities of Bello, Medellín, and Itagüí (Antioquia department) ranged from 80% to 97% [26,27,28,29,30,31,193]. Because biological vector-control technologies may generate concerns or misinformation regarding genetically modified organisms, environmental effects, or perceived experimentation, communication should clearly explain the biological mechanism involved, distinguish *Wolbachia*-based mosquito replacement from genetically modified mosquito technologies, describe available evidence on safety and effectiveness, and provide accessible mechanisms for communities to raise questions and participate in implementation decisions [190,194,195].

Integrated vector management programs are also incorporating other innovative tools. These include autocidal gravid ovitraps, spatial repellents, sterile insect techniques, incompatible insect techniques (IIT), enhanced entomological surveillance systems, and new insecticide formulations designed to improve residual activity and address operational challenges associated with insecticide resistance. Unlike *Wolbachia*-mediated population replacement, IIT exploits *Wolbachia*-induced cytoplasmic incompatibility by releasing infected males to suppress target mosquito populations rather than establish *Wolbachia* and reduce vector competence within the population. Microencapsulated formulations are one such approach, potentially allowing for prolonged release and persistence of active compounds while improving chemical control performance under specific field conditions. Nevertheless, these approaches should be distinguished from *Wolbachia*-mediated population replacement, for which disease-endpoint evidence, long-term persistence after establishment, implementation protocols, operational experience, and economic evaluations are substantially more advanced. Accordingly, newer tools should continue to be assessed within an IVM framework according to local resistance profiles, ecological conditions, operational capacity, epidemiological effectiveness, cost-effectiveness, and sustainability [196,197,198].

Increasingly, experts emphasize that no single intervention can control dengue transmission in complex endemic environments. In high-transmission urban settings, *Wolbachia*-mediated population replacement can reduce the force of infection at the community level and thereby complement vaccination while providing area-wide protection that does not depend on individual vaccine uptake or repeated administration. Integrated prevention, however, should not imply that all available interventions have equivalent levels of evidence or operational readiness. Scale-up decisions should prioritize tools with demonstrated epidemiological effectiveness, established safety, operational feasibility, and capacity for sustained implementation, while other promising technologies continue evaluation. Community engagement remains particularly important because household-level practices strongly influence mosquito breeding and exposure risk. Educational campaigns, risk communication, and local participation in source reduction activities remain key elements of sustainable prevention programs [199,200]. However, community engagement should not transfer the principal responsibility for vector control to households. Effective and sustained source reduction also depends on reliable municipal waste collection, secure water supply, environmental sanitation, inspection of public and private premises, and, where appropriate within national and local regulatory frameworks, enforcement measures to reduce persistent mosquito-breeding conditions [201,202].

Climate change adds complexity to vector control efforts. Rising temperatures, altered rainfall patterns, and extreme weather events are expanding mosquito habitats and modifying seasonal transmission dynamics across the Americas. As a result, prevention programs must become increasingly adaptive and climate-sensitive, incorporating environmental monitoring and predictive tools into operational planning [203,204,205].

Future vector-control programs should prioritize combinations of interventions selected according to local entomological, epidemiological, environmental, and operational conditions. Novel biological technologies, strengthened surveillance, community-based approaches, and conventional control measures are most likely to achieve sustained impact when implemented within an integrated and continuously evaluated framework rather than as isolated interventions [32,145,206].

## 7. Toward an Integrated Regional Response to Dengue in the Americas

The unprecedented dengue epidemics observed in the Americas during recent years have demonstrated that isolated or fragmented interventions are insufficient to address the growing complexity of arboviral transmission in the region. A One Health framework provides a useful conceptual basis for this integrated response by recognizing the interconnectedness of human health, vector ecology, animal and environmental health, and the social and ecological systems that shape arboviral transmission. Although dengue is primarily maintained through human–*Aedes* transmission in urban settings, environmental determinants, climate, land use, water and waste management, vector ecology, human mobility, and the broader circulation of other arboviruses with zoonotic or sylvatic components strongly influence its prevention. Applying a One Health perspective therefore encourages collaboration across public health, clinical medicine, entomology, veterinary sciences, environmental health, urban planning, climatology, and community sectors [47,62,123,207,208,209].

At the regional level, this approach is also consistent with PAHO’s Integrated Management Strategy for Dengue (*Estrategia de Gestión Integrada* [EGI]-Dengue), which promotes coordinated action across epidemiological surveillance, laboratory diagnosis, patient care, integrated vector management, environmental management, communication, and operational response (https://iris.paho.org/items/e39936a2-26ab-479c-a374-5ec09b4ecb9e) (accessed on 1 August 2026).

Dengue is no longer solely a vector-borne disease problem but rather a multidimensional public health challenge influenced by environmental change, social inequities, urbanization, population mobility, healthcare capacity, and climate variability [60,63,210]. Consequently, the region requires integrated, multisectoral, and sustainable response strategies capable of addressing both the immediate burden of epidemics and the structural conditions that facilitate transmission [211,212,213].

A coordinated regional response must prioritize strengthening surveillance systems, improving laboratory capacity, harmonizing reporting standards, and enabling rapid information sharing between countries. Regional harmonization should move beyond general recommendations toward an agreed-upon minimum surveillance framework that defines standardized case classifications, laboratory confirmation criteria, severity categories, age and sex strata, hospitalization and mortality indicators, serotype reporting, socioeconomic and geographic indicators where available, and other essential epidemiological variables. Countries should progressively adopt interoperable digital reporting systems and standardized data dictionaries that allow for timely aggregation and comparison of national and subnational data across the Americas. The heterogeneity of surveillance methodologies and case definitions across the Americas continues to limit the comparability of epidemiological data and complicates regional preparedness efforts. PAHO, together with national health authorities and regional reference laboratories, could facilitate the development of a consensus-based normative framework that establishes minimum reporting standards, recommended notification timelines, laboratory and genomic surveillance criteria, and periodic data-quality assessments. Such a framework should preserve countries’ regulatory sovereignty while progressively aligning surveillance definitions and indicators, thereby improving cross-border outbreak detection, regional risk assessment, and evaluation of intervention effectiveness. Regional benchmarking and periodic evaluation of adherence to these standards could provide a practical mechanism for identifying surveillance gaps and supporting targeted technical assistance [214,215].

Expanding the use of integrated digital surveillance systems, genomic monitoring, climate-informed forecasting models, and real-time outbreak-detection tools may substantially improve early-warning capacities and support more timely public health interventions [86,143,146]. Table 2 summarizes the principal components of integrated dengue prevention and control strategies currently being implemented or evaluated in the Americas. Financial sustainability should be considered a core component of this integrated response. Dengue imposes substantial direct medical costs through outpatient care, emergency visits, hospitalization, critical care, diagnostics, and epidemic response, while indirect costs related to absenteeism, productivity losses, caregiving, and premature mortality further increase its societal burden. Earlier regional analyses estimated the annual economic cost of dengue in Latin America and the Caribbean at more than US$3 billion. Still, these estimates predate the unprecedented epidemics of 2023–2024 and therefore likely underestimate the current burden. Economic evaluations of dengue vaccination have suggested that vaccination can avert a meaningful proportion of illness-related costs and may be cost-effective in high-transmission settings. In contrast, economic analyses of *Wolbachia*-based interventions indicate that their substantial initial deployment costs may be offset over time by reductions in dengue cases, hospitalizations, and outbreak-response expenditures. Rather than evaluating vaccination, vector control, surveillance, and clinical preparedness independently, future economic evaluations should increasingly assess integrated intervention portfolios and identify the combinations that maximize health gains and disability-adjusted life years averted within locally available budgets. These comparisons should explicitly account for the different operational and financing profiles of available interventions. Vaccination may require repeated procurement, age-targeted delivery, high and sustained coverage, cold-chain capacity, and long-term budget commitments. In contrast, *Wolbachia*-mediated population replacement can provide area-wide protection across age groups and may achieve long-term persistence after successful establishment, potentially reducing the need for recurrent releases. However, continued entomological monitoring and occasional supplementary deployment may be required. Comparative policy analyses should therefore evaluate not only short-term cost-effectiveness but also duration of protection, recurrent versus upfront costs, population coverage, implementation capacity, equity, and long-term sustainability when determining the optimal mix of interventions [216,217].

The increasing simultaneous deployment of vaccination and *Wolbachia*-mediated population replacement creates an important new challenge for evaluating dengue-control programs. Mato Grosso do Sul, Brazil, provides an illustrative setting, with large-scale *Wolbachia* deployment in Campo Grande and mass dengue vaccination implemented in Dourados. As these and other interventions are progressively introduced within the same broader epidemiological settings, changes in dengue incidence, hospitalization, and severe disease may become increasingly difficult to attribute to a single intervention. Future impact evaluations should therefore be designed prospectively to distinguish, where possible, the independent, additive, and potentially synergistic effects of vaccination and *Wolbachia* while accounting for differences in intervention timing and coverage, population mobility, baseline transmission intensity, serotype circulation, secular trends, and other concurrent control measures. Factorial, stepped-wedge, controlled interrupted time-series, synthetic-control, and other quasi-experimental designs may be useful, depending on implementation conditions. This emerging scenario also creates an opportunity to determine whether combining population-level reduction in force of infection through *Wolbachia* with individual protection through vaccination produces greater and more durable population impact than either intervention alone [22,27,90,91,185,187,189,194,217].

Financing mechanisms are equally important. Regional pooled procurement mechanisms such as the PAHO Revolving Fund can strengthen purchasing power and support more predictable, equitable vaccine access. At the same time, GAVI’s 2026–2030 strategy anticipates potential support for dengue vaccination in eligible lower-income and selected middle-income countries. Nevertheless, most countries in the Americas will continue to depend substantially on domestic financing, making affordability, budget impact, sustained political commitment, and equitable allocation central considerations when scaling up vaccination and innovative vector-control technologies [106,218,219,220].

Many countries in the Americas remain highly dependent on constrained public-sector budgets. At the same time, newer interventions, including vaccination, advanced surveillance systems, genomic monitoring, and innovative vector-control technologies, may require substantial initial and recurrent investments. Sustainable implementation therefore requires medium- and long-term financing strategies, prioritization based on local disease burden and cost-effectiveness, and mechanisms that prevent access to effective interventions from becoming concentrated in better-resourced countries, regions, or populations [221,222]. In parallel, countries should establish explicit implementation pathways for incorporating *Wolbachia*-mediated population replacement into routine vector-control programs where local epidemiological and operational conditions are suitable. These pathways should address regulatory authorization, local production or sustainable supply arrangements, workforce training, community co-design and engagement, release planning, quality assurance, entomological monitoring, and long-term epidemiological evaluation. Brazil provides an important regional precedent for this transition from research-led deployment to a nationally supported public health intervention. Embedding these components within national vector-control structures may facilitate program continuity, reduce dependence on short-term project funding, and support more equitable and sustainable scale-up [195,223].

At the same time, vaccination strategies should be incorporated into broader prevention frameworks adapted to local epidemiological realities. New-generation dengue vaccines offer an important opportunity to reduce severe disease and hospitalizations, particularly in highly endemic areas. However, vaccine implementation should be accompanied by strengthened vector control programs, community participation, public communication strategies, and continuous pharmacovigilance systems to maximize effectiveness and maintain public confidence [16,17,19,20,116,121,224].

Equity considerations are also fundamental for sustainable dengue control. The burden of dengue disproportionately affects populations living in social and economic disadvantage, particularly in areas characterized by inadequate sanitation, precarious housing, irregular water supply, deficient solid waste collection and disposal, and limited access to timely healthcare. These conditions increase opportunities for *A. aegypti* breeding while simultaneously reducing households’ and communities’ capacity to implement and sustain preventive measures. Poverty therefore acts not only as a marker of vulnerability but also as a structural barrier to effective dengue prevention, early diagnosis, and access to appropriate care. Sex and gender, occupation, migration status, ethnicity, age, disability, and caregiving roles also shape social vulnerability. They can influence exposure to mosquito vectors, healthcare-seeking behavior, access to preventive interventions, and illness outcomes. However, dengue surveillance and programmatic reporting in the region do not consistently disaggregate these dimensions sufficiently. Routine analysis of incidence, hospitalization, severe disease, mortality, vaccine uptake, and access to vector-control interventions by sex, age, socioeconomic status, ethnicity, and other locally relevant equity variables would help identify populations experiencing disproportionate risk and support more targeted interventions. Addressing these inequities requires long-term investment in urban infrastructure, reliable water and waste-management services, environmental management, primary healthcare systems, and social protection policies, together with prevention strategies explicitly designed to reach underserved populations [225,226,227].

Regional cooperation between governments, international organizations, academic institutions, and research centers is increasingly necessary to strengthen preparedness for future epidemics. Collaborative initiatives focused on training, research, innovation, and technical cooperation can help disseminate best practices and accelerate the implementation of evidence-based interventions across countries. In addition, the growing impact of climate change on arboviral transmission underscores the need to adopt adaptive, climate-resilient public health strategies [228,229]. Health-system adaptation should extend beyond epidemiological forecasting to the resilience of physical infrastructure and essential services. Facilities in areas vulnerable to floods, hurricanes, extreme rainfall, heat waves, drought, or other climate-related hazards should maintain contingency plans for uninterrupted electricity, safe water, laboratory operations, cold-chain capacity, communications, supply chains, and referral and emergency transport systems. Hospitals and primary-care networks should also develop scalable surge plans for abrupt increases in febrile illness, including flexible bed capacity, pre-positioned fluids and essential medicines, rapid diagnostic capability, and mechanisms to redistribute personnel and supplies between affected areas. Climate-informed preparedness should further integrate meteorological early-warning systems with entomological surveillance and healthcare demand forecasting, enabling intensified vector control, community communication, vaccination where applicable, and clinical preparedness before transmission peaks. These measures matter because extreme weather events can simultaneously increase vector breeding opportunities, disrupt water and waste-management services, displace populations, and impair access to healthcare [208,230,231].

Ultimately, the future of dengue prevention in the Americas will depend on countries’ ability to move beyond reactive epidemic responses toward integrated, preventive, and sustainable regional frameworks grounded in One Health principles and combining surveillance, vaccination, vector control, clinical preparedness, environmental management, scientific innovation, and social participation into a unified public health strategy [37,145].

## 8. Limitations

This narrative review has several limitations. It originated from the themes and discussions of the Dengue Summit and was subsequently expanded through a narrative literature review; therefore, topic selection and emphasis may partly reflect Summit participants’ expertise, perspectives, and priorities. Although we searched multiple bibliographic databases and institutional sources, we did not use a systematic review methodology, a predefined search strategy, a formal study-selection process, or a risk-of-bias assessment. Consequently, we may have overlooked relevant evidence, and we cannot rule out selection and citation bias. Several authors are active contributors to dengue research, policy, clinical management, vaccination, and vector-control initiatives in the Americas, which may have led to citation of work by members of the author group. We attempted to mitigate these limitations by incorporating evidence from independent studies, international organizations, national programs, and diverse countries and disciplines. Accordingly, interpret this review as an expert-informed narrative synthesis rather than an exhaustive systematic assessment of the evidence.

## 9. Conclusions

Dengue remains one of the most important and rapidly evolving public health threats in the Americas. The unprecedented epidemics observed in 2023–2024 exposed the region’s growing vulnerability to large-scale arboviral outbreaks and highlighted the limitations of traditional control strategies amid accelerating urbanization, climate variability, social inequities, and weakened public health infrastructure. At the same time, these epidemics also stimulated important scientific, technological, and operational advances that are reshaping the regional response to dengue.

Recent progress in dengue vaccination has created new opportunities to reduce severe disease and hospitalizations in endemic settings. Implementation experiences from countries such as Brazil, Peru, and Argentina demonstrate that large-scale vaccination programs are operationally feasible when supported by strong political commitment, technical capacity, and community engagement. Nevertheless, vaccination alone will not be sufficient to control dengue transmission in highly endemic environments, reinforcing the need for integrated prevention frameworks that combine immunization with vector control, surveillance, environmental management, and clinical preparedness.

Innovations in surveillance systems, predictive modeling, and digital epidemiology are also transforming countries’ capacity to detect outbreaks earlier and respond more efficiently. Integrating climate information, genomic surveillance, artificial intelligence, and real-time data analysis provides valuable tools to strengthen preparedness and support evidence-based public health decision-making. Similarly, advances in clinical management guidelines and regional capacity-building initiatives are improving recognition and treatment of severe dengue, particularly in critical care settings.

Vector control remains an essential pillar of dengue prevention, but its role is evolving beyond conventional approaches. *Wolbachia*-mediated population replacement should now be regarded as an evidence-based and operationally mature intervention rather than merely an emerging alternative. Randomized, large-scale implementation studies have shown substantial reductions in dengue, including reductions exceeding 90% in Colombian cities where stable *Wolbachia* establishment was achieved. Its standardized implementation methods and expanding programmatic use support a transition from repeated demonstration projects toward sustained integration within national vector-control strategies, accompanied by rigorous monitoring and coordination with surveillance, clinical preparedness, environmental management, and appropriately targeted vaccination. Its potential multipathogen effect against other *Aedes*-borne arboviruses may further increase its public-health value, although population-level effectiveness beyond dengue remains less well established.

The discussions at the 1st Dengue Summit at the Global Health Conference of the Americas emphasized the urgent need for stronger regional cooperation and coordinated action. Future preparedness efforts must prioritize equity, resilience, scientific innovation, and multisectoral collaboration to confront the expanding threat of dengue and other arboviral diseases. Building integrated regional frameworks that combine prevention, surveillance, research, vaccination, vector control, and healthcare strengthening will be essential to reducing the future burden of dengue in the Americas and improving public health security across the region.

## Figures and Tables

**Table 1:** Dengue cases, incidence, severity, and lethality in the Americas, 2025.

Country/Subregion	Total Cases	Incidence Rate *	Laboratory Confirmed	%Laboratory Confirmed	Severe	%Severe	Deaths	CFR%	Population × 1000
Canada	0	0.00	0	0	0	0.00	0	0.000	40,126
United States of America	1153	0.33	1153	100.00	25	2.17	0	0.000	347,275
North America	1153	0.30	1153	100.00	25	2.17	0	0.000	387,401
Belize	2797	662.80	1537	54.95	0	0.00	0	0.000	422
Costa Rica	6019	116.83	3367	55.94	0	0.00	0	0.000	5152
El Salvador	5833	91.64	203	3.48	5	0.09	3	0.051	6365
Guatemala	48,278	258.35	1002	2.08	6	0.01	6	0.012	18,687
Honduras	17,368	157.82	478	2.75	230	1.32	8	0.046	11,005
Mexico	145,251	110.08	22,001	15.15	772	0.53	84	0.058	131,946
Nicaragua	5702	81.38	916	16.06	0	0.00	0	0.000	7007
Panama	19,809	433.36	16,262	82.09	122	0.62	28	0.141	4571
Central America Isthmus and Mexico	251,057	135.59	45,766	18.23	1135	0.45	129	0.051	185,155
Bolivia	38,028	302.27	1357	3.57	203	0.53	3	0.008	12,581
Colombia	125,119	234.20	86,881	69.44	1241	0.99	124	0.099	53,425
Ecuador	37,840	206.90	37,840	100.00	367	0.97	65	0.172	18,289
Peru	39,211	113.41	34,260	87.37	193	0.49	56	0.143	34,576
Venezuela **	28,110	98.58	N/A	N/A	N/A	N/A	N/A	N/A	28,516
Andean Subregion ***	240,198	162.97	160,338	66.75	2004	0.83	248	0.103	147,387
Argentina	17,648	38.49	8111	45.96	47	0.27	13	0.074	45,851
Brazil	3,834,357	1801.76	1,469,805	38.33	2595	0.07	1826	0.048	212,812
Chile	0	0.00	0	0.00	0	0.00	0	0.000	19,859
Paraguay	31,656	451.39	1374	4.34	0	0.00	1	0.003	7013
Uruguay	7	0.21	7	100.00	0	0.00	0	0.000	3384
Southern Cone	3,883,668	1344.21	1,479,297	38.09	2642	0.07	1840	0.047	288,919
Cuba	30,692	280.63	441	1.44	770	2.51	19	0.062	10,937
Dominican Republic	2011	17.46	326	16.21	19	0.94	0	0.000	11,520
Puerto Rico	3319	102.60	2542	76.59	129	3.89	2	0.060	3235
Latin Caribbean	36,022	140.21	3309	9.19	918	2.55	21	0.058	25,692
Anguilla	3	21.43	3	100.00	0	0.00	0	0.000	14
Antigua and Barbuda	15	15.96	10	66.67	0	0.00	0	0.000	94
Aruba	14	12.96	3	21.43	0	0.00	0	0.000	108
Bahamas	0	0.00	0	0.00	0	0.00	0	0.000	403
Barbados	178	63.12	1	0.00	0	0.00	0	0.000	282
Bermuda	1	1.56	0	0.00	0	0.00	0	0.000	64
Bonaire, Saint Eustatius and Sabia	0	0.00	0	0.00	0	0.00	0	0.000	31
Cayman Islands	0	0.00	0	0.00	0	0.00	0	0.000	75
Curaçao	0	0.00	0	0.00	0	0.00	0	0.000	185
French Guiana	318	101.60	318	100.00	0	0.00	0	0.000	313
Grenada	217	185.47	0	0.00	5	2.30	0	0.000	117
Guadeloupe	5214	1397.86	44	0.84	0	0.00	0	0.000	373
Guyana	70,056	8389.94	15,234	21.75	2305	3.29	7	0.010	835
Haiti	0	0.00	0	0.00	0	0.00	0	0.000	11,906
Jamaica	602	21.22	1	0.17	0	0.00	0	0.000	2837
Martinique	1280	376.47	250	19.53	0	0.00	0	0.000	340
Montserrat	3	75.00	3	100.00	0	0.00	0	0.000	4
Saint Barthelemy	20	181.82	3	15.00	0	0.00	0	0.000	11
Saint Kitts and Nevis	11	23.91	5	45.45	0	0.00	0	0.000	46
Saint Lucia	47	26.11	47	100.00	0	0.00	0	0.000	180
Saint Martin	214	891.67	2	0.93	0	0.00	0	0.000	24
Saint Vincent and the Grenada	8	8.08	8	100.00	0	0.00	0	0.000	99
Sint Maarten	8	18.60	8	100.00	0	0.00	0	0.000	43
Suriname	1313	205.48	268	20.41	0	0.00	2	0.152	639
Trinidad and Tobago	73	4.83	3	4.11	0	0.00	0	0.000	1511
Turks and Caicos Islands	1	2.17	0	0.00	0	0.00	0	0.000	46
Virgin Islands (UK)	46	117.95	46	100.00	0	0.00	0	0.000	39
Virgin Islands (US)	47	55.95	47	100.00	0	0.00	0	0.000	84
Non-Latin Caribbean	79,689	384.92	16,304	20.46	2310	2.90	9	0.011	20,703
The Americas	**4,491,787**	**425.66**	**1,706,167**	**37.98**	**9034**	**0.2**	**2247**	**0.05**	**1,055,257**

* Cases per 100,000 pop. ** Recent epidemiological data available from the Ministry of Health of Venezuela. https://mpps.gob.ve/boletines-epidemiologicos/. Access date: 1 August 2026. *** Not including Venezuela. Subregional groupings follow the PAHO dengue surveillance classification used to analyze dengue in the Americas, which may differ from the geographic groupings used in other PAHO data platforms. Accordingly, Mexico is included in the Central America and Mexico subregion, whereas Brazil is included in the Southern Cone. Bold indicates totals for the continent. N/A, not available. CFR, case fatality rate. UK, United Kingdom. US, United States of America.

**Table 2:** Integrated Strategies for Dengue Prevention and Control in the Americas.

Strategy	Main Components	Advantages	Limitations/Challenges	Examples in the Americas
Vaccination	Use of tetravalent dengue vaccines, targeted immunization campaigns, integration into national immunization programs, and pharmacovigilance systems	Reduction in severe dengue, hospitalizations, and mortality; complementary to vector control	High cost, unequal access, vaccine hesitancy, logistical barriers, uncertainties regarding long-term effectiveness	TAK-003 implementation in Brazil and Argentina; targeted vaccination strategies in Colombia
Integrated vector management	Source reduction, larval control, environmental sanitation, insecticide application, entomological surveillance, and community participation	Broad applicability, immediate reduction in mosquito density, adaptable to local contexts	Insecticide resistance, operational fatigue, sustainability challenges, and dependence on community adherence	National vector control programs across Latin America and the Caribbean
*Wolbachia*-based interventions	*Wolbachia*-mediated *A. aegypti* population replacement to reduce vector competence and interrupt arboviral transmission	Area-wide protection across age groups; strong disease-endpoint evidence for dengue; self-sustaining after successful establishment; reduced reliance on insecticides; favorable long-term cost-effectiveness	Requires initial production and deployment capacity, regulatory coordination, intensive community engagement, quality assurance, and post-release entomological and epidemiological monitoring; substantial upfront investment is concentrated during implementation	Large-scale deployment in Bello, Medellín, and Itagüí, Colombia, associated with >90% reduction in notified dengue incidence in areas with successful *Wolbachia* establishment; national programmatic scale-up and expanding biofactory capacity in Brazil
Community engagement and risk communication	Educational campaigns, household participation in source reduction, public communication, and school-based interventions	Improves prevention practices, strengthens vaccine confidence, and increases the sustainability of interventions	Misinformation, inconsistent participation, social inequities, and communication fatigue	Community mobilization initiatives during dengue outbreaks in multiple endemic countries
Digital and integrated surveillance systems	Real-time dashboards, automated reporting platforms, laboratory integration, geographic information systems, outbreak monitoring	Earlier outbreak detection, improved situational awareness, and evidence-based decision-making	Data quality heterogeneity, limited infrastructure in low-resource settings, and delayed reporting	PAHO regional surveillance platforms and national digital reporting systems
Climate-informed forecasting and mathematical modeling	Integration of climate data, predictive analytics, artificial intelligence, and outbreak forecasting models	Supports preparedness planning, early warning systems, and optimized resource allocation	Uncertainty in predictive accuracy, dependence on high-quality data, and limited technical capacity	Forecasting initiatives in Brazil, Colombia, and regional collaborative projects
Clinical preparedness and standardized management	Evidence-based clinical guidelines, ICU protocols, healthcare worker training, triage systems	Reduces severe disease mortality, improves patient outcomes, and standardizes care	Limited healthcare capacity, shortage of trained personnel, and unequal access to critical care	Regional severe dengue management guidelines and training initiatives
Genomic and laboratory surveillance	Viral sequencing, serotype monitoring, molecular diagnostics, RT-PCR capacity strengthening	Improved understanding of transmission dynamics, identification of emerging strains, and serotype shifts	Limited laboratory infrastructure, high costs, unequal regional access	National reference laboratories and collaborative genomic surveillance networks in the Americas

Note: The table summarizes evidence and implementation experiences discussed and referenced in the corresponding sections of the document.

**Table 3:** Comparative Characteristics and Efficacy of Dengue Vaccines Relevant to the Americas [22,90,91].

Characteristic	TAK-003 (Qdenga^®^) (Takeda^®^)	CYD-TDV (Dengvaxia^®^) (Sanofi Pasteur^®^)	Butantan-DV^®^ (Instituto Butantan)
**Platform**	Live-attenuated tetravalent vaccine. Attenuated DENV-2 provides the genomic backbone; the DENV-1, DENV-3, and DENV-4 components are chimeric viruses expressing the corresponding serotype surface antigens.	Recombinant live-attenuated tetravalent vaccine using the yellow fever 17D virus backbone expressing DENV-1–4 structural antigens.	Live-attenuated tetravalent, TV003-derived vaccine developed from the NIAID dengue vaccine platform.
**Schedule**	2 doses, months 0 and 3.	3 doses, months 0, 6, and 12.	Single dose.
**Requirement for previous dengue infection**	No requirement for documented previous infection and no routine pre-vaccination serological screening required under the WHO-recommended programmatic strategy.	Yes. Vaccination should be restricted to individuals with evidence of previous dengue infection because vaccination of dengue-naïve individuals can increase their subsequent risk of severe dengue/hospitalization.	No pre-vaccination screening requirement in the Brazilian indication; approved for individuals with or without previous dengue exposure.
**Population evaluated in pivotal efficacy trial**	20,099 children/adolescents aged 4–16 years in eight dengue-endemic countries in Latin America and Asia.	CYD15: 20,869 participants aged 9–16 years in Brazil, Colombia, Honduras, Mexico, and Puerto Rico.	16,235 participants aged 2–59 years were enrolled in Brazil; 10,259 received the vaccine, and 5976 received placebo in the long-term analysis.
**Overall efficacy against symptomatic virologically confirmed dengue**	73.3% (95%CI 66.5–78.8) through approximately 18 months; 61.2% (56.0–65.8) cumulatively through approximately 4.5 years.	60.8% (95%CI 52.0–68.0) in the per-protocol Latin American phase 3 analysis; 64.7% (58.7–69.8) in the intention-to-treat analysis.	65.0% (95%CI 57.8–71.0) cumulatively during 5 years of follow-up.
**Efficacy in baseline seropositive participants**	76.1% (68.5–81.9) in the 18-month analysis; 64.2% (58.4–69.2) cumulatively at 4.5 years.	In CYD15, efficacy in the baseline-seropositive subgroup was 83.7% (62.2–93.7).	77.1% (67.6–83.9) over 5 years in dengue-experienced participants.
**Efficacy in baseline seronegative participants**	66.2% (49.1–77.5) at approximately 18 months; 53.5% (41.6–62.9) cumulatively at 4.5 years. Protection in seronegative participants is established primarily against DENV-1 and DENV-2.	Lower efficacy and an unfavorable longer-term benefit–risk profile in dengue-naïve recipients; consequently, current use requires evidence of previous infection.	58.9% (48.0–67.6) over 5 years in dengue-naïve participants.
DENV-1 efficacy *	69.8% (95%CI 54.8–79.9) in the 18-month phase 3 serotype analysis.	50.3% in CYD15, Latin America.	73.0% (95%CI 64.3–79.7) over 5 years.
DENV-2 efficacy *	95.1% (89.9–97.6) in the 18-month phase 3 analysis.	42.3% in CYD15, Latin America.	55.7% (42.3–66.1) over 5 years.
DENV-3 efficacy *	48.9% (27.2–64.1) overall in the 18-month phase 3 analysis. Long-term efficacy was demonstrated in baseline-seropositive participants but was not demonstrated in baseline-seronegative participants.	74.0% in CYD15, Latin America.	Not estimable. No DENV-3 cases occurred during the phase 3 efficacy follow-up, so serotype-specific VE could not be estimated.
DENV-4 efficacy *	51.0% (−69.4 to 85.8) in the 18-month phase 3 analysis; estimate highly imprecise because of few cases. At 4.5 years, protection was demonstrated among baseline-seropositive participants, whereas insufficient DENV-4 cases prevented assessment in baseline-seronegative participants.	77.7% in CYD15, Latin America.	Not estimable. No DENV-4 cases occurred during the efficacy follow-up.
**Efficacy against hospitalization/severe outcomes**	Cumulative efficacy against hospitalized virologically confirmed dengue at 4.5 years: 84.1% (77.8–88.6); 85.9% (78.7–90.7) in baseline seropositives and 79.3% (63.5–88.2) in baseline seronegatives.	In CYD15, efficacy against hospitalization was 80.3% and against severe virologically confirmed dengue was 95.5% during the active surveillance period. These estimates preceded recognition of the longer-term excess risk among seronegative vaccinees.	Efficacy against the combined endpoint of dengue with warning signs or severe dengue over 5 years: 80.5% (50.8–92.4).
**WHO position**	WHO recommends programmatic use primarily in children aged 6–16 years living in settings with high dengue transmission intensity; 2-dose schedule 3 months apart. WHO cautions against programmatic use in low-to-moderate transmission settings until the DENV-3/DENV-4 efficacy–risk profile among seronegative individuals is better established. WHO prequalified TAK-003 in May 2024.	Use is restricted to persons with evidence of previous dengue infection. Its programmatic role has become very limited.	No WHO programmatic recommendation or WHO prequalification was identified in the sources reviewed through 9 August 2026.

* Serotype-specific efficacy estimates are derived from different pivotal trials and follow-up periods and should not be interpreted as head-to-head comparisons. Regulatory and programmatic status reflects information reviewed through 9 August 2026. CI, confidence interval; CYD-TDV, chimeric yellow fever–dengue tetravalent dengue vaccine; DENV, dengue virus; DENV-1, dengue virus serotype 1; DENV-2, dengue virus serotype 2; DENV-3, dengue virus serotype 3; DENV-4, dengue virus serotype 4; NIAID, National Institute of Allergy and Infectious Diseases; TAK-003, Takeda tetravalent dengue vaccine candidate 003; TV003, tetravalent live-attenuated dengue vaccine candidate TV003; VE, vaccine efficacy; WHO, World Health Organization. Butantan Institute (São Paulo, Brazil).

**Table 4:** Regulatory and Programmatic Status of Dengue Vaccines in Selected Countries of the Americas.

Country	TAK-003 (Qdenga^®^)	CYD-TDV (Dengvaxia^®^)	Butantan-DV
**Brazil**	ANVISA registration since March 2023 for 4–60 years, regardless of previous dengue; two doses 3 months apart. Incorporated into public vaccination strategies.	ANVISA still listed Dengvaxia as a registered vaccine in its April 2026 update, indicated for previously infected persons aged 6–45 years.	ANVISA published registration in December 2025 for ages 12–59, single dose. The national vaccination strategy was temporarily suspended on 8 June 2026 while rare unexpected post-vaccination events are investigated; registration itself was not withdrawn.
**Argentina**	ANMAT approved TAK-003 in April 2023 for persons ≥4 years of age, regardless of prior dengue. Targeted vaccination has subsequently been implemented in selected jurisdictions/populations.	No major current national programmatic role identified.	Not approved/implemented in the sources reviewed.
**Colombia**	Registered/evaluated by INVIMA; Colombia began phased inclusion in the National Vaccination Schedule in 2026, initially targeting 9-year-olds/fourth-graders in prioritized municipalities, with two doses 3 months apart.	No current routine national program identified.	Not approved/implemented in the sources reviewed.
**Peru**	TAK-003 is being used in the public dengue vaccination strategy in prioritized regions; the 2026 strategy includes persons aged 10–20 years and selected frontline personnel.	No current routine national program identified.	Not approved/implemented in the sources reviewed.
**Main programmatic advantage**	No requirement for serological prescreening; substantial protection against hospitalization; growing real-world implementation experience in the Americas.	Demonstrated protection in previously dengue-infected individuals and extensive long-term clinical experience.	Single-dose regimen; broad age range; demonstrated efficacy in both dengue-experienced and dengue-naïve participants.
**Principal limitation/current concern**	Heterogeneous efficacy according to serotype and baseline serostatus, particularly uncertainty regarding DENV-3 and DENV-4 in seronegative individuals; completion of a two-dose series is required.	Need to document previous dengue infection; historical safety concern in seronegative recipients; manufacturer has discontinued production, markedly limiting future programmatic relevance.	No phase 3 efficacy estimate is yet available for DENV-3 or DENV-4 because these serotypes did not circulate sufficiently during the efficacy trial; additionally, the Brazilian vaccination strategy has been temporarily halted since June 2026 pending investigation of a post-marketing safety signal.

The table summarizes the principal evidence and considerations discussed and referenced in the corresponding sections of the document. ANMAT, National Administration of Drugs, Foods and Medical Devices (Administración Nacional de Medicamentos, Alimentos y Tecnología Médica); ANVISA, Brazilian Health Regulatory Agency (Agência Nacional de Vigilância Sanitária); CYD-TDV, chimeric yellow fever–dengue tetravalent dengue vaccine; DENV, dengue virus; DENV-3, dengue virus serotype 3; DENV-4, dengue virus serotype 4; INVIMA, National Institute for Food and Drug Surveillance (Instituto Nacional de Vigilancia de Medicamentos y Alimentos); TAK-003, Takeda tetravalent dengue vaccine candidate 003.

## Data Availability

The original contributions presented in this study are included in the article. Further inquiries can be directed to the corresponding author.

## Supplementary Materials

The following supporting information can be downloaded at: https://www.mdpi.com/article/10.3390/pathogens15101019/s1, Table S1. Comparative Epidemiological, Clinical, Diagnostic, and Preventive Characteristics of Major Arboviral Diseases in the Americas.
