# Supplementary material for: Toward an Integrated Response to Arboviral Threats in the Americas: Perspectives from the Dengue Summit of the Global Health Conference of the Americas 2025
Source: Pathogens. 2026 Sep 27;15(10):1019. doi: 10.3390/pathogens15101019 (PMC13708027; doi:10.3390/pathogens15101019)
Supplement: Supplementary file 1 [file pathogens-15-01019-s001.zip › pathogens-4545551-supplementary.pdf]

Supplementary Table S1. Comparative Epidemiological, Clinical, Diagnostic, and Preventive Characteristics of Major Arboviral Diseases in the Americas.

Disease/virus	Principal vector(s) and epidemiology in the Americas	Incubation	Predominant clinical presentation / useful clues	Severe, atypical, or persistent manifestations	Pregnancy / congenital considerations	Characteristic laboratory findings	Preferred laboratory diagnosis	Human vaccine status/development	Key prevention and clinical considerations
**Dengue (DENV-1–4)**	Primarily *Aedes aegypti; A. albopictus* contributes in some settings. Widespread endemic/hyperendemic transmission throughout Latin America and the Caribbean, with expanding risk in subtropical and previously low-transmission areas. All four serotypes circulate in the Americas.	Usually 5–7 days	Abrupt fever, severe headache, retro-orbital pain, marked myalgia/arthralgia, nausea, vomiting, and macular/maculo-papular rash. The clinical course may evolve through the febrile, critical, and recovery phases. Progressive thrombocytopenia, rising hematocrit, and warning signs favor dengue over other uncomplicated arboviral febrile illnesses.	Plasma leakage, shock, severe bleeding, myocarditis, hepatitis/acute liver injury, encephalopathy/encephalitis, and multiorgan dysfunction. Post-acute fatigue, weakness, and other persistent manifestations may occur.	Vertical/perinatal transmission is possible, especially with maternal infection close to delivery; adverse pregnancy outcomes have been reported.	Leukopenia, thrombocytopenia, hemoconcentration during plasma leakage, hyponatremia, and increased AST/ALT.	During acute illness: NAAT/RT-PCR + IgM, or NS1 antigen + IgM. Molecular/NS1 testing is particularly useful during the first week. IgM becomes increasingly useful after several days; consider flavivirus serologic cross-reactivity.	Multiple vaccines now exist, including CYD-TDV, TAK-003 and Butantan-DV, with substantially different indications, serostatus requirements, schedules and national regulatory status; see Tables 3 and 4 of the review.	Integrated vector management, source reduction, personal mosquito protection and vaccination where indicated. In suspected disease, avoid aspirin/NSAIDs until dengue is excluded, recognize warning signs early, and use judicious isotonic fluid management.
**Chikungunya (CHIKV)**	Mainly *Aedes aegypti* and *A. albopictus*. Established in the Americas since its introduction in 2013, with periodic major outbreaks and ongoing transmission.	Usually 3–7 days; range 1–12	Sudden high fever plus intense, frequently disabling bilateral and symmetric polyarthralgia/polyarthritis, often affecting hands, wrists, ankles and feet. Rash, headache, myalgia, and conjunctivitis can occur. Severe joint pain is among the strongest clinical clues distinguishing it from dengue and Zika.	Persistent or relapsing arthralgia/arthritis lasting months to years; rarely myocarditis, hepatitis, nephritis, uveitis/retinitis, meningoencephalitis, myelitis, GBS and other neurologic disease. Severe disease is more likely in neonates, older adults, and persons with comorbidities.	Perinatal transmission is well documented, particularly when maternal viremia occurs around delivery, and can cause severe neonatal disease.	Lymphopenia is characteristic; thrombocytopenia may occur but is generally less prominent than in dengue; elevated creatinine and transaminases can occur.	RT-PCR/NAAT during approximately the first week; serology thereafter. CHIKV-specific IgM should ideally be confirmed with neutralizing antibodies when necessary.	VIMKUNYA, a recombinant virus-like particle vaccine, is FDA-licensed for persons aged≥12 years in the USA. The live attenuated IXCHIQ had its U.S. biologics license suspended in August 2025 and its accelerated approval withdrawn in January 2026 due to safety concerns; its regulatory status differs in other jurisdictions.	Personal protection against Aedes, vector control and vaccination of appropriately selected persons where available/recommended. Initial analgesia should favor acetaminophen/paracetamol until dengue has been reasonably excluded. Persistent inflammatory arthropathy may require longer-term clinical management.
**Zika (ZIKV)**	Predominantly *Aedes aegypti*; transmission also occurs sexually and vertically. The explosive 2015–2016 epidemic has subsided, but autochthonous transmission persists at low levels in parts of the Americas.	3–14 days	Approximately 80% are asymptomatic. Symptomatic illness is usually mild: maculopapular rash, low-grade fever, nonpurulent conjunctivitis, arthralgia, myalgia and headache. Prominent rash/conjunctivitis with relatively mild systemic illness favors Zika.	GBS, neuropathy, myelitis, encephalitis/encephalopathy, uveitis, and rarely severe thrombocytopenia.	Major distinguishing feature. Maternal infection can cause congenital Zika syndrome, including microcephaly, cortical/cerebra	Usually nonspecific; thrombocytopenia can occur but marked thrombocytopenia should prompt consideration of dengue or severe/atypical disease.	NAAT is preferred for confirming acute infection; serum and sometimes urine can be tested depending on timing and clinical context. Serology is complicated by substantial flavivirus cross-reactivity, especially with dengue	No licensed vaccine. Vaccine development remains active, but no product is currently available for clinical prevention.	Daytime mosquito-bite prevention; prevention of sexual transmission through condoms/abstinence according to exposure guidance; particular protection of pregnant persons and those planning pregnancy. Dengue should be excluded before NSAID use.

Disease/virus	Principal vector(s) and epidemiology in the Americas	Incubation	Predominant clinical presentation / useful clues	Severe, atypical, or persistent manifestations	Pregnancy / congenital considerations	Characteristic laboratory findings	Preferred laboratory diagnosis	Human vaccine status/development	Key prevention and clinical considerations
					l atrophy, intracranial abnormalities, ocular lesions, fetal growth abnormalities, miscarriage, and other adverse outcomes.		and previous flavivirus vaccination. Pregnancy requires specific testing algorithms.		
**Yellow fever (YFV)**	Sylvatic transmission involving *Haemagogus*/*Sabethes* mosquitoes and nonhuman primates predominates in the Americas. Endemic risk is concentrated in tropical South America. Increased activity was documented in 2025–2026, including human cases and epizootics.	Usually 3–6 days	Acute fever, chills, severe headache, prominent myalgia/back pain, nausea/vomiting, and prostration. Relative bradycardia (Faget sign) can occur. A subset improves briefly and then develops a toxic phase characterized by jaundice, hemorrhage, and renal/hepatic dysfunction.	Fulminant hepatitis, coagulopathy, gastrointestinal hemorrhage, acute kidney injury, shock, and multiorgan failure; case-fatality among severe cases is high.	Routine live vaccine use is generally avoided during pregnancy, except when the risk of exposure outweighs the potential risks of vaccination. Maternal infection can be severe, but congenital disease is not the defining clinical feature as in Zika.	Leukopenia early; thrombocytopenia, prolonged PT/aPTT, rising bilirubin, marked AST/ALT elevation, and renal abnormalities in severe disease.	RT-PCR/NAAT early, typically during the first week; IgM/neutralizing antibodies later. Flavivirus cross-reactivity and prior YF vaccination complicate serology. A negative RT-PCR result does not rule out yellow fever when obtained outside the viremic window. Tissue RT-PCR, histopathology, and immunohistochemistry are useful in fatal cases.	Highly effective live-attenuated 17D vaccine available. A single dose generally provides long-lasting/lifelong protection for most persons.	Vaccination is the cornerstone; mosquito-bite prevention remains complementary. In endemic settings, surveillance of nonhuman-primate epizootics provides important early warning.
**Oropouche virus disease (OROV)**	Primarily transmitted by the biting midge *Culicoides paraensis*; some mosquitoes can participate. Historically centered in the Amazon basin but expanded markedly from late 2023 onward into new areas of South America and the Caribbean.	1–10 days	Abrupt fever, severe headache, chills, myalgia, arthralgia, retro-orbital pain, photophobia, dizziness, nausea/vomiting, and rash. Recurrence of a similar febrile syndrome days or weeks later is characteristic and is frequently reported.	A small minority develop meningitis/encephalitis or other neurologic disease; hemorrhagic manifestations and rare GBS have been described.	Emerging major concern. Vertical transmission has been associated with fetal death/stillbirth and congenital abnormalities, including CNS defects, although the magnitude and full causal	Leukopenia/lymphopenia, mildly increased transaminases and CRP; thrombocytopenia can occur.	RT-PCR days 0–7; if PCR-negative around days 6–7, PRNT can be added; PRNT after day 7. Serum and CSF can be tested. Pregnancy/congenital disease requires expanded testing, potentially including maternal serum/urine, amniotic fluid, placenta, fetal tissues, cord blood and neonatal specimens.	No licensed vaccine and no specific antiviral therapy.	Protection against biting midges and mosquitoes, vector control around human habitation, surveillance in newly affected areas and special counseling of pregnant travelers/residents. Until dengue is excluded, avoid NSAIDs in acute undifferentiated illness.

Disease/virus	Principal vector(s) and epidemiology in the Americas	Incubation	Predominant clinical presentation / useful clues	Severe, atypical, or persistent manifestations	Pregnancy / congenital considerations	Characteristic laboratory findings	Preferred laboratory diagnosis	Human vaccine status/development	Key prevention and clinical considerations
					spectrum remain under active investigation.				
**Mayaro virus disease (MAYV)**	Primarily *Haemagogus* spp. mosquitoes in a sylvatic cycle involving nonhuman primates. Reported in South America, Central America and the Caribbean; Brazil has documented the largest number of recognized cases. Forest exposure is a major epidemiologic clue.	1–14 days	Acute fever, headache, chills, myalgia, rash and prominent arthralgia, especially wrists, fingers, ankles and toes. Clinically resembles chikungunya; rash often develops several days after onset.	Persistent arthralgia may last months and occasionally up to a year. Severe disease is uncommon.	Data on vertical transmission and congenital disease are much more limited than for Zika, chikungunya, or Oropouche; no established congenital syndrome.	Leukopenia; occasionally mild thrombocytopenia.	Molecular detection during acute viremia; serology/neutralization thereafter. Testing is generally restricted to reference/public-health laboratories, and alphavirus serologic cross-reactivity must be considered.	No licensed vaccine. Vaccine research exists, but clinical development remains far less advanced than for dengue or chikungunya.	Mosquito avoidance, particularly in forested areas; occupational/travel counseling; vector surveillance. Clinically, consider alongside chikungunya when severe or persistent arthralgia dominates.
**West Nile virus disease (WNV)**	*Culex* spp. mosquitoes; enzootic bird–mosquito cycle. Established throughout much of North America and present elsewhere in the Americas. Humans and horses are typically dead-end hosts. Transfusion and transplantation transmission are rare but recognized.	2–14 days, sometimes longer in immunoco mpromised patients	About 80% asymptomatic. About 20% develop West Nile fever: fever, fatigue, headache, myalgia, arthralgia, rash and GI symptoms. Less than 1% develop neuroinvasive disease.	Meningitis, encephalitis and a characteristic acute flaccid myelitis/poliomyelitis-like syndrome; tremor, myoclonus and parkinsonian features may occur. Older age and immunosuppression strongly increase risk. Neurologic sequelae may persist long term.	Congenital infection is possible but appears rare; unlike Zika, no consistent congenital syndrome has been established.	Routine blood findings are nonspecific. Neuroinvasive disease: CSF lymphocytic pleocytosis, sometimes neutrophilic early; increased protein and generally normal glucose.	WNV IgM in serum and/or CSF is the first-line diagnostic test. IgM generally becomes detectable 3–8 days after onset. PRNT may be required for confirmation because of flavivirus cross-reactivity. RT-PCR is particularly useful in immunocompromised patients.	No licensed human vaccine, although effective veterinary vaccines exist.	Mosquito control, personal protection, blood-donor screening and organ-donor risk mitigation. Neurologic disease requires supportive hospital/ICU care.
**Eastern equine encephalitis (EEEV)**	Mosquito-bird enzootic cycle, particularly in freshwater hardwood swamps in eastern North America; several mosquito species bridge transmission to humans/equids. Rare but among the most severe mosquito-borne encephalitides in the Americas.	4–10 days	Most infections are asymptomatic or present as nonspecific febrile illness. Neuroinvasive disease causes fever, headache, meningismus, confusion, focal neurologic deficits, seizures, and rapidly progressive coma.	Approximately 30% case-fatality among severe cases; around half of survivors of severe disease can have substantial neurologic disability.	No well-established congenital syndrome; pregnancy-specific data are limited.	CSF may initially show neutrophil-predominant pleocytosis, later lymphocytic predominance, elevated protein levels, and normal glucose levels.	EEEV-specific IgM in serum or CSF, followed by neutralizing-antibody confirmation. RT-PCR has low sensitivity in routine immunocompetent cases but may be useful in immunocompromised patients or selected CSF/tissue specimens.	No licensed human vaccine. Investigational multivalent EEEV/WEEV/VEEV vaccines have reached early-phase human clinical evaluation.	Mosquito avoidance and community vector control. No specific antiviral; rapid recognition of encephalitis, seizure control, airway protection, and management of intracranial complications are critical.
**Venezuelan equine encephalitis (VEEV)**	Mosquito-borne alphavirus with enzootic and epizootic subtypes.	Usually 2–5 days	Abrupt febrile illness with severe headache, chills,	Encephalitis is uncommon overall but occurs particularly	Fetal infection and congenital	Leukopenia/lymph openia are	During early acute disease, RT-PCR/virus	No licensed human vaccine. TC-83/C-84	Human mosquito protection, plus equine surveillance,

Disease/virus	Principal vector(s) and epidemiology in the Americas	Incubation	Predominant clinical presentation / useful clues	Severe, atypical, or persistent manifestations	Pregnancy / congenital considerations	Characteristic laboratory findings	Preferred laboratory diagnosis	Human vaccine status/development	Key prevention and clinical considerations
	Epizootic strains can efficiently amplify in equids, producing large equine and human epidemics in northern South America, Central America, and Mexico. Major historical outbreaks have occurred in Colombia and Venezuela.		malaise, myalgia, back pain, anorexia, nausea/vomiting; pharyngitis may occur. Clinical illness can resemble influenza or other arboviral febrile illnesses.	in children and older adults; seizures and neurologic sequelae may occur. Humans can develop sufficiently high viremia to participate in mosquito-mediated amplification during epizootics.	abnormalities have been described, although the evidence base is far smaller than for Zika.	common during acute disease; other abnormalities are nonspecific.	detection may be useful because viremia can be substantial; VEEV-specific IgM and neutralizing antibodies are used subsequently. Testing generally requires specialized/reference laboratories.	have historically been used as investigational products in highly exposed personnel. Newer DNA, VLP, and multivalent EEE/WEE/VEE candidates have undergone phase 1 evaluation.	vaccination, and movement control, are particularly important because epizootic equids can amplify transmission.
**St. Louis encephalitis (SLEV)**	Culex spp. mosquitoes and birds form the enzootic cycle. Historically important across the Americas, including the USA and parts of Latin America; outbreaks can overlap geographically with WNV.	4–14 days	Most infections are asymptomatic. Symptomatic disease begins with fever, headache, dizziness, nausea and weakness. Neuroinvasive disease presents with meningitis or encephalitis; older age strongly predicts severity.	Altered mental status, seizures, speech abnormalities, paresis/paralysis, movement disorders and cranial nerve palsies. Case-fatality among recognized encephalitis cases is approximately 5–20%; persistent tremor, gait/speech impairment or cognitive/behavioral sequelae can occur.	No established congenital syndrome.	Neuroinvasive disease: typically lymphocytic CSF pleocytosis, increased CSF protein and usually normal glucose; routine peripheral laboratory findings are nonspecific.	SLEV-specific IgM in serum and/or CSF, with positive results confirmed by neutralization because cross-reactivity with WNV and other flaviviruses can be substantial. Molecular testing may be considered, especially in immunocompromised patients.	No licensed human vaccine.	Personal mosquito protection and integrated Culex control. Clinical management is supportive; neurologic disease may require hospitalization and intensive supportive care.

ALT, alanine aminotransferase; AST, aspartate aminotransferase; CHIKV, chikungunya virus; CNS, central nervous system; CRP, C-reactive protein; CSF, cerebrospinal fluid; DENV, dengue virus; EEEV, Eastern equine encephalitis virus; GBS, Guillain–Barré syndrome; IgM, immunoglobulin M; MAYV, Mayaro virus; NAAT, nucleic acid amplification test; NSAIDs, nonsteroidal anti-inflammatory drugs; OROV, Oropouche virus; PRNT, plaque-reduction neutralization test; RT-PCR, reverse transcription polymerase chain reaction; SLEV, St. Louis encephalitis virus; VEEV, Venezuelan equine encephalitis virus; WNV, West Nile virus; YFV, yellow fever virus; ZIKV, Zika virus.

Clinical interpretation: Clinical features overlap substantially among arboviral infections, particularly during the first days of illness. No individual sign or symptom reliably establishes the etiologic diagnosis; epidemiological exposure, local circulation, illness timing, pregnancy status, and laboratory testing should be interpreted together.
Laboratory interpretation: Molecular testing is generally most informative during early viremia for dengue, chikungunya, Zika, yellow fever, Oropouche, Mayaro, and Venezuelan equine encephalitis, whereas IgM testing of serum and/or cerebrospinal fluid is central for neuroinvasive flaviviruses such as West Nile and St. Louis encephalitis viruses and for Eastern equine encephalitis virus. Serological cross-reactivity, particularly among flaviviruses, may require confirmation using plaque-reduction neutralization testing. The optimal specimen and diagnostic window vary according to the virus, host immune status, and stage of illness.
Analgesic management: In patients presenting with an undifferentiated acute arboviral syndrome in a dengue-risk area, aspirin and other nonsteroidal anti-inflammatory drugs should generally be avoided until dengue has been reasonably excluded, given the potential hemorrhagic risk.
Selection of diseases: The 10 infections were selected based on regional burden, outbreak potential, clinical severity, geographic relevance, and emerging importance; the list is not intended as a formal ranking.

Relevant Sources for Table S1.

Source	URL
Chikungunya clinical signs	https://www.cdc.gov/chikungunya/hcp/clinical-signs/index.html
Chikungunya diagnosis	https://www.cdc.gov/chikungunya/hcp/diagnosis-testing/index.html
Dengue clinical care	https://www.cdc.gov/dengue/hcp/clinical-care/index.html
Dengue clinical signs	https://www.cdc.gov/dengue/hcp/clinical-signs/index.html
Dengue diagnosis and testing	https://www.cdc.gov/dengue/hcp/diagnosis-testing/index.html
Eastern equine encephalitis clinical signs	https://www.cdc.gov/eastern-equine-encephalitis/hcp/clinical-signs/index.html
Eastern equine encephalitis diagnosis	https://www.cdc.gov/eastern-equine-encephalitis/hcp/diagnosis-testing/index.html
FDA VIMKUNYA	https://www.fda.gov/vaccines-blood-biologics/vimkunya
Mayaro clinical diagnosis	https://www.cdc.gov/mayaro-virus/hcp/clinical-diagnosis-treatment/index.html
Mayaro data/maps	https://www.cdc.gov/mayaro-virus/data-maps/index.html
Oropouche clinical overview	https://www.cdc.gov/oropouche/hcp/clinical-overview/index.html
Oropouche reporting/testing	https://www.cdc.gov/oropouche/php/reporting/index.html
Oropouche symptoms	https://www.cdc.gov/oropouche/symptoms/index.html
PAHO Oropouche	https://www.paho.org/en/topics/oropouche-virus-disease
St. Louis encephalitis clinical signs	https://www.cdc.gov/sle/hcp/clinical-signs/index.html
St. Louis encephalitis diagnosis	https://www.cdc.gov/sle/hcp/diagnosis-testing/index.html
West Nile clinical signs	https://www.cdc.gov/west-nile-virus/hcp/clinical-signs/index.html
West Nile diagnosis	https://www.cdc.gov/west-nile-virus/hcp/diagnosis-testing/index.html
WHO dengue vaccines Q&A	https://www.who.int/news-room/questions-and-answers/item/dengue-vaccines
WHO integrated arbovirus clinical guideline	https://www.who.int/publications/i/item/9789240111110
WHO yellow fever fact sheet	https://www.who.int/news-room/fact-sheets/detail/yellow-fever
WHO Zika fact sheet	https://www.who.int/news-room/fact-sheets/detail/zika-virus
Yellow fever clinical diagnosis	https://www.cdc.gov/yellow-fever/hcp/clinical-diagnosis/index.html
Zika clinical signs	https://www.cdc.gov/zika/hcp/clinical-signs/index.html
Zika diagnosis	https://www.cdc.gov/zika/hcp/diagnosis-testing/index.html
